# Antimicrobial and Antiviral Nanofibers Halt Co‐Infection Spread via Nuclease‐Mimicry and Photocatalysis

**DOI:** 10.1002/advs.202309590

**Published:** 2024-04-22

**Authors:** Jieran Yao, Zhenhong Luo, Jiaying Lin, Na Meng, Jiangna Guo, Hui Xu, Rongwei Shi, Linhui Zhao, Jiateng Zhou, Feng Yan, Bin Wang, Hailei Mao

**Affiliations:** ^1^ Department of Critical Care Medicine Zhongshan Hospital Fudan University Shanghai 200032 China; ^2^ College of Chemistry Chemical Engineering and Materials Science Soochow University Suzhou 215123 China; ^3^ Department of Plastic and Reconstructive Surgery Shanghai Ninth People's Hospital Shanghai Jiao Tong University School of Medicine Shanghai 200011 China; ^4^ School of Material and Chemical Engineering Tongren University Tongren 554300 China

**Keywords:** antibiotic resistance genes, antimicrobial and antiviral nanofibers, artificial nuclease, photodynamic therapy, poly(ionic liquid), viral nucleic acids

## Abstract

The escalating spread of drug‐resistant bacteria and viruses is a grave concern for global health. Nucleic acids dominate the drug‐resistance and transmission of pathogenic microbes. Here, imidazolium‐type poly(ionic liquid)/porphyrin (PIL‐P) based electrospun nanofibrous membrane and its cerium (IV) ion complex (PIL‐P‐Ce) are developed. The obtained PIL‐P‐Ce membrane exhibits high and stable efficiency in eradicating various microorganisms (bacteria, fungi, and viruses) and decomposing microbial antibiotic resistance genes and viral nucleic acids under light. The nuclease‐mimetic and photocatalytic mechanisms of the PIL‐P‐Ce are elucidated. Co‐infection wound models in mice with methicillin‐resistant *S. aureus* and hepatitis B virus demonstrate that PIL‐P‐Ce integrate the triple effects of cationic polymer, photocatalysis, and nuclease‐mimetic activities. As revealed by proteomic analysis, PIL‐P‐Ce shows minimal phototoxicity to normal tissues. Hence, PIL‐P‐Ce has potential as a “green” wound dressing to curb the spread of drug‐resistant bacteria and viruses in clinical settings.

## Graphical abstract

Poly(ionic liquid)/porphyrin/cerium‐based nanofibrous membranes with antimicrobial and antiviral activities are synthesized. The PIL‐P‐Ce membranes effectively eradicate multiple microorganisms (bacteria, fungi, and viruses) and decompose microbial ARGs and viral DNA/RNA under 650 nm light. These membranes have potential clinical applications as a “green” wound dressing to prevent the spread of infections.

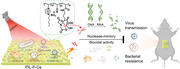

## 1 Introduction

The surge of fast‐spreading drug‐resistant bacteria and viruses poses a severe global health threat. Nucleic acids dominate the pathogenicity, drug‐resistance, and transmissibility of microbes. Bacteria can dodge drugs via antibiotic resistance genes (ARGs), essentially DNA, thus highly stable and degradation‐resistant.^[^ ^1^, ^2^ ^]^ Even post bacteria elimination, ARGs can endure the environment, transferring horizontally among microorganisms.^[^ ^3^, ^4^ ^]^ ARGs fundamentally drive the emergence and spread of antimicrobial resistance. Viral nucleic acid is vital for assessing virus replication and disease infectivity.^[^ ^5^ ^]^ Obviously, it is crucial to inactivate ARGs and viral nucleic acids while killing microbes.

Artificial nucleases employ multinuclear metal complexes like Ce(IV) to simulate natural enzymes' hydrolysis of phosphodiester bonds, generally used against bacterial biofilms.^[^ ^6^, ^7^, ^8^, ^9^ ^]^ Our report marks the first integration of nuclease‐mimic into tangible antimicrobial materials, effectively eliminating ARGs.^[^ ^10^ ^]^ The material's weak antiviral effect could be due to the virus's unique structure, as viral nucleic acids are protected by a protein capsid, potentially covered by a lipid envelope. Thus, other component must be introduced to enhance the antiviral effect with cationic polymers.

Poly(ionic liquid), notable for their designability and stability, can disrupt bacterial cell walls and lipid membranes.^[^ ^11^, ^12^ ^]^ Photodynamic therapy (PDT) uses photosensitizers to produce reactive oxygen species (ROS) that can damage lipids, proteins, nucleic acids.^[^ ^13^, ^14^ ^]^ Combining of PIL, PDT and artificial nucleases could bolster antimicrobial and antiviral effects, blocking the spread of ARG and infectious nucleic acids. No such reports exist to our knowledge.

Here, imidazolium‐type poly(ionic liquid)/porphyrin (PIL‐P) based electrospun nanofibrous membrane and its cerium (IV) ion complex (PIL‐P‐Ce) were synthesized. The antimicrobial (against *S. aureus*, *E. coli*, and *C. albicans*), antiviral (against hepatitis B and C viruses) effects, degradation activities toward ARGs and viral nucleic acids, were investigated under different light conditions. The nuclease‐mimetic and photocatalytic mechanisms of PIL‐P‐Ce were also explored. In vivo efficacies of PIL‐P‐Ce against methicillin‐resistant *S. aureus* (MRSA) and HBV co‐infections in mouse skin wounds were tested. Phototoxicity to normal wound tissues was assessed through proteomics. Based on these results, PIL‐P‐Ce is a promising new antimicrobial and antiviral dressings with intrinsic nuclease activity to combat infection spread.

## 2 Results and Discussion

### 2.1 Fabrication and Characterization of PIL‐P‐Ce

**Figure 1:**
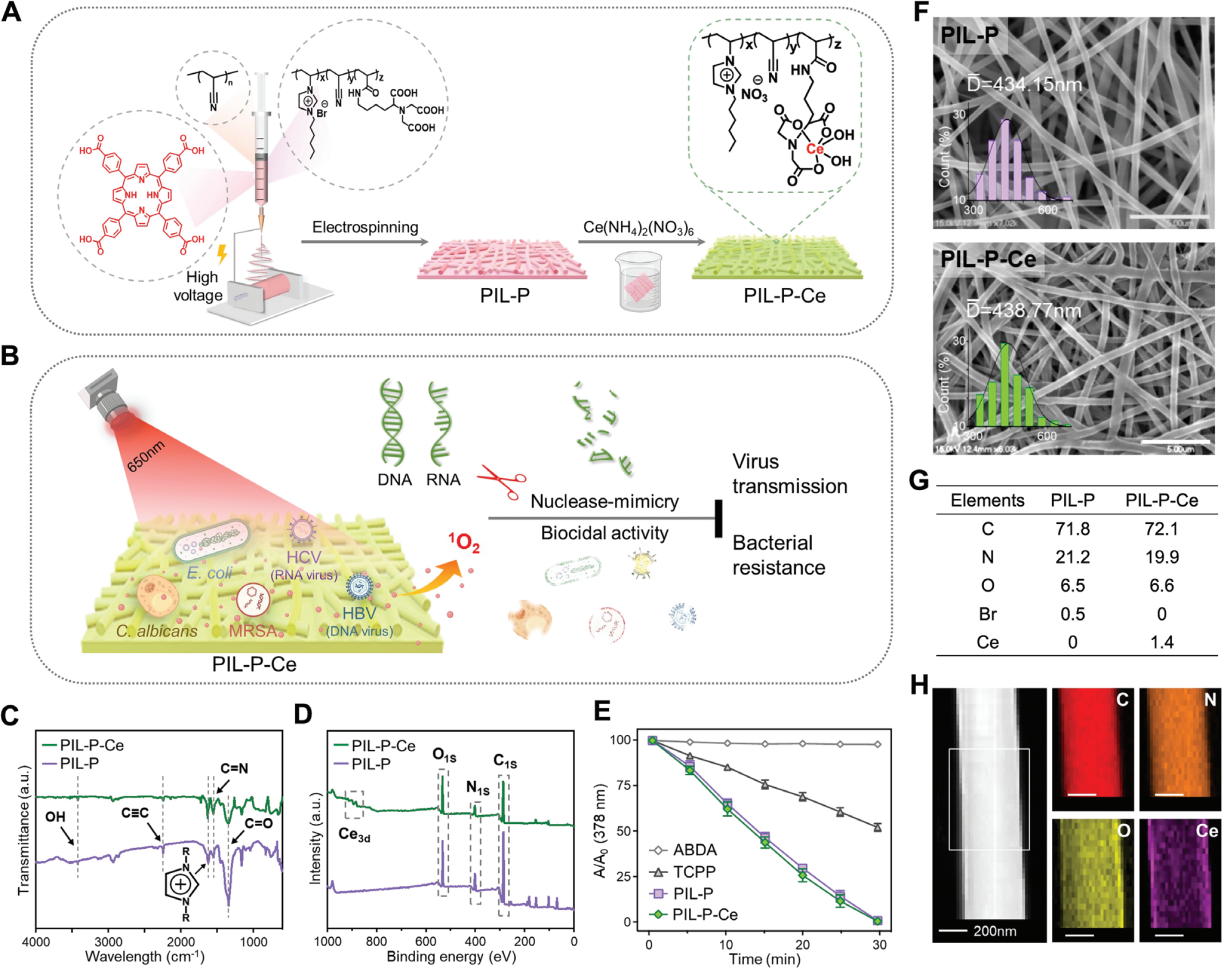
Strategy of antimicrobial and antiviral nanofibers with nuclease‐mimetic and photocatalytic activities for blocking the spread of infections. A) Imidazolium‐type poly(ionic liquid)/porphyrin based electrospun nanofibrous membrane (PIL‐P) and its cerium (IV) ion complex (PIL‐P‐Ce) were synthesized. B) PIL‐P‐Ce membrane, combining the activities of cationic polymer and photocatalytic and nuclease‐mimetic moieties, can eradicate pathogens (bacteria, fungi, and viruses) and decompose microbial drug‐resistant genes and viral DNA/RNA under 650 nm light, thereby blocking both bacterial resistance and viral transmission. C,D) FT‐IR and XPS spectra of PIL‐P and PIL‐P‐Ce. E) ABDA decomposition rates with TCPP or PIL‐P‐based membranes under 650 nm light (3.5 mW cm^−2^), with A_0_ and A being initial and measured ABDA absorbance at 378 nm over time. F,G) SEM images, fiber diameter distributions, and EDS data of PIL‐P‐based membranes. Scale bar, 5 µm. H) Element mapping images of PIL‐P‐Ce nanofibers under TEM. Scale bar, 200 nm.

Imidazolium‐type PIL was mixed with photosensitizer tetracarboxyphenylporphyrin (TCPP) to fabricate PIL‐P membranes via electrospinning, subsequently complexed with Ce^4+^ to obtain PIL‐P‐Ce (**Figure** **1**A). PIL‐P‐Ce was designed to kill various pathogens and degrade microbial ARGs and viral nucleic acids under 650 nm light (Figure 1B). The ionic liquid monomer, [HVIm][Br] and metal ligand, AANTA, were synthesized and polymerized into P(IL‐co‐AANTA‐co‐AN) using free‐radical copolymerization (Figure S1, Supporting Information). The chemical structure of two monomers and their copolymer were confirmed by ^1^H NMR spectra (Figures S2–S4, Supporting Information). The resulting PIL‐P membranes were fabricated via high‐voltage electrospinning of a solution containing P(IL‐co‐AANTA‐co‐AA), polyacrylonitrile (PAN) and photosensitizer TCPP in dimethylformamide (DMF), followed by immersion in a Ce(NH_4_)_2_(NO_3_)_6_ solution to complex with Ce^4+^ ions (Figure 1A).

The obtained PIL‐P and PIL‐P‐Ce membranes are uniformly spun, soft, and planar films that can be cut to any size. PIL‐P is pinkish due to TCCP mixture, while PIL‐P‐Ce appears yellow due to Ce^4+^ ion complexation. Fourier transform infrared (FT‐IR) spectroscopy confirmed successful incorporation of AANTA, imidazolium cation, cyano group, and TCPP into the fibers (Figure 1C). PIL‐P‐Ce's reduced 1722 cm^−1^ peak indicated successful Ce^4+^ ion coordination. X‐ray photoelectron spectroscopy (XPS) shows the presence of Ce element in PIL‐P‐Ce at ≈900 eV and confirm O, N, and C elements are common in both samples (Figure 1D). To measure the amount of ROS produced by the PIL‐P‐based membranes, we used the water‐soluble probe ABDA, which can be specifically oxidized by singlet oxygen (^1^O_2_).^[^ ^15^, ^16^ ^]^ Each membrane sample (1 cm^2^) was incubated with ABDA solution under 650 nm light (3.5 mW cm^−2^) for varying durations, and the decrease in absorbance at 378 nm was used to estimate ^1^O_2_ yield. Figure 1E shows that both PIL‐P and PIL‐P‐Ce membranes rapidly and efficiently produced ^1^O_2_, further confirming their photocatalytic activity. Scanning electron microscopy (SEM) displays that both PIL‐P‐based membranes have fibers with a uniform diameter of ≈435 nm, with PIL‐P‐Ce fibers slightly less homogeneous due to water swelling during Ce^4+^ coordination (Figure 1F). Energy dispersive spectroscopy (EDS) confirms the presence of Ce element in PIL‐P‐Ce, suggesting successful coordination (Figure 1G). The uniform Ce signals on PIL‐P‐Ce nanofiber in the transmission electron microscopy (TEM) element mapping images further support this (Figure 1H). Ce content in PIL‐P‐Ce, measured by inductively coupled plasma mass spectroscopy (ICP‐MS), is ≈4.03 wt.%. These results confirm the successful synthesis of PIL‐P‐Ce nanofibrous membranes.

The PIL‐P and PIL‐P‐Ce membranes display high air permeability (≈65%), significant water absorption rates (≈175% and 135%), and low water swelling rates (≈10% and 8%), with no further changes observed after a week in a PBS solution (Figure S5A,B, Supporting Information). Thermogravimetric analysis (TGA) indicates their high thermal stabilities, making them suitable for sterilization (Figure S5C, Supporting Information). Additionally, they exhibit relatively low protein adsorption (≈19 µg cm^−2^), and bigger contact angles (124° and 129°), suggesting good antifouling capabilities (Figure S5D, Supporting Information). These physical properties make the materials potentially beneficial for in vivo wound treatment. PIL‐P‐based membranes' biocompatibility was evaluated using human erythrocytes, fibroblasts, and hepatoma cells.^[^ ^17^, ^18^ ^]^ They met hemolysis and cell growth standards, regardless of light treatment. Although minor decrease of relative growth rate (RGR, 78–83%) occurred with light exposure, cell morphology remained stable (Figure S6, Supporting Information). This demonstrates the safety of PIL‐P‐based membranes in vitro.

### 2.2 Nuclease‐Mimetic and Photocatalytic Activities

The hydrolytic performance of PIL‐P‐Ce on phosphodiester bonds was first tested using BNPP.^[^ ^19^ ^]^ BNPP, containing a phosphodiester bond, is cleaved into p‐nitrophenol by Ce complexes, producing a yellow solution with peak absorption at 400 nm (Figure S7A, Supporting Information). The color change was observed in PIL‐P‐Ce with light for 30 min or not, indicating the decomposition of BNPP. PIL‐P did not show any 400 nm absorbance, while PIL‐P‐Ce rose linearly during 4 h co‐incubation, especially when exposed to light (Figure S7B, Supporting Information). This validate Ce^4+^ and porphyrin's role in phosphodiester bond hydrolysis. Ce^4+^ ions act as artificial nucleases by directly breaking phosphodiester bond and making it susceptible to nucleophilic attack,^[^ ^6^, ^20^ ^]^ while porphyrins facilitate the hydrolysis process through the generation of ^1^O_2_ as a nucleophilic species.^[^ ^21^, ^22^ ^]^ The enzymatic‐like property of PIL‐P‐Ce was evaluated by monitoring the initial cleavage velocity of varying BNPP concentrations. Both curves with and without light sharply increased before 0.5 mm BNPP, then stabilized at a high level (Figure S7C, Supporting Information). PIL‐P‐Ce's *K*_m_ values were calculated as 0.23 mm (with light) and 0.27 mm (without light), indicating enhanced nuclease‐like activity in light due to porphyrin's photocatalysis. In recycling experiments conducted under light to assess PIL‐P‐Ce's stability for enzymatic hydrolysis, PIL‐P‐Ce retained over 80% decomposition efficiency after five cycles and ≈70% after eight cycles, demonstrating its stability and reusability despite slight efficiency reduction from repeated light exposure (Figure S7D, Supporting Information).

**Figure 2:**
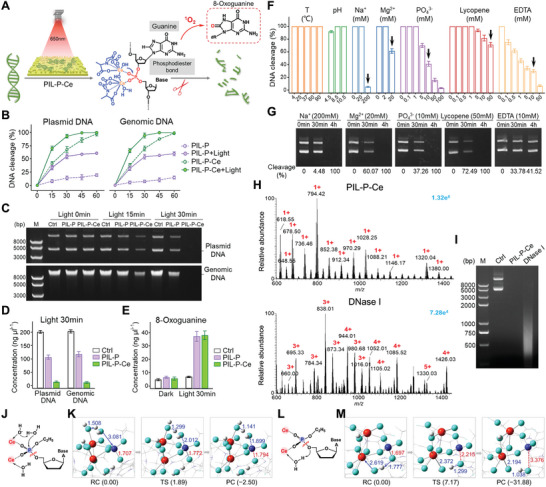
Nuclease‐mimetic and photocatalytic activities for DNA cleavage. A) Diagram showing DNA decomposition by PIL‐P‐Ce under light. B) Cleavage efficiency of *E. coli* plasmid and genomic DNA by tested membranes, with/without light for 30 min. C) Electrophoretograms depicting light‐exposed DNA levels with tested membranes. D) DNA concentration changes post‐irradiation for 30 min. E) 8‐oxoguanine production in DNA samples exposed to membranes with/without light. F,G) DNA cleavage efficiencies of PIL‐P‐Ce using UV absorbance and gel electrophoresis under various conditions with light for 30 min and subsequently without light till 4 h. Figure 2G illustrates the conditions indicated by arrows in Figure 2F. H,I) High‐resolution mass spectra and electrophoresis images of DNA degradation products by PIL‐P‐Ce and DNase I. Red numbers indicate charge states; blue numbers provide relative ion abundances. PIL‐P‐Ce was lighted for 30 min, and DNase I reacted for the same duration. J–M) Ce(IV) complex‐mediated DNA hydrolysis mechanisms, with corresponding structures of reaction complex (RC), transitional state (TS) and product complex (PC) optimized via density functional theory. Ce^4+^ bound to water/hydroxide molecules assists either the leaving group (J,K) or nucleophilic attack (L,M) to activate P─O hydrolytic cleavage of phosphodiesters in DNA analogues. Element colors: Ce (red), P (blue), O (azure), H (white). Indicators: reaction directions (single arrows), P─O bond cleavage (red wavy lines), P─O bond length (red dotted lines), adjacent atom distances (blue dash lines), other interatomic distances (in Å), relative energies (kcal mol^−1^ in brackets).

The effectiveness of PIL‐P‐Ce was further evaluated on DNA, with two damage targets: phosphodiester bonds and guanine. Through combined nuclease‐like and photocatalytic activities, the phosphodiester bond is damaged, while guanine is oxidized to 8‐oxoguanine by porphyrin (**Figure** **2**A).^[^ ^23^ ^]^ DNA degradation by PIL‐P‐Ce was tested on *E. coli* genomic and plasmid DNA. Even without light, PIL‐P‐Ce could almost completely degrade DNA in 60 min, while PIL‐P achieved ≈10% cleavage (Figure 2B). Ce^4+^ ions in PIL‐P‐Ce were instrumental, and the minor PIL‐P effect can be attributed to electrostatic interaction between imidazolium cations and DNA. With light exposure, DNA degradation was faster. DNA concentration in PIL‐P‐Ce group dropped to ≈20 ng µL^−1^ within 30 min (≈90% decomposition), while over half of DNA in PIL‐P group remained (Figure 2B–D). As microbial ARGs can exist in both chromosomal and plasmid DNA, this suggests potential ARG degradation by PIL‐P‐Ce. Porphyrin's photocatalytic activity was unaffected by Ce^4+^ complexation, as ≈40 ng µL^−1^ of 8‐oxoguanine was produced by both films under light (Figure 2E). These results indicate that PIL‐P‐Ce can coordinate cationic polymer, photocatalytic and nuclease‐mimetic activities for DNA destruction.

The nuclease‐like activity of PIL‐P‐Ce was assessed under various conditions to elucidate its mechanism. Its activity remained stable across a wide temperature range of 4–90 °C and pH of 4.5–10.5, demonstrating superior stability over natural enzymes. Low concentrations of Na^+^, Mg^2+^, and PO_4_^3−^ had no substantial impact, but high ion concentrations downregulated PIL‐P‐Ce's activity. However, this inhibition was counteracted over 4 h, suggesting ions and phosphates at physiological concentrations would not influence its efficiency. The antioxidant lycopene could temporarily disrupt PIL‐P‐Ce photocatalysis, but DNA damage was compensated by ongoing intrinsic nuclease activity. Conversely, the chelating agent EDTA caused an irreversible loss of nuclease‐mimetic activity by binding to Ce^4+^, even over 4 h (Figure 2F,G; Figure S8, Supporting Information). This highlights the key role of complexed Ce^4+^ in PIL‐P‐Ce's DNA cleavage capability.

Using high‐resolution mass spectrometry (MS), we compared the DNA cleavage efficiency of PIL‐P‐Ce and DNase I. Given that the average mass of a base pair (bp) is ≈650 Da, we calculated the size of DNA fragments in samples based on the mass‐to‐charge ratio (m/z) in MS spectra. Figure 2H shows the PIL‐P‐Ce group's product peaks were single‐charged and distributed between 600 and 1400 Da, indicating that PIL‐P‐Ce can decompose bacterial plasmid DNA into 1–2 bp fragments after 30 min of light exposure. Comparatively, the DNase I group mostly had 3–5+ peaks, with a 1:100 enzyme‐to‐substrate ratio for the same reaction time, suggesting the DNA decomposition products of natural enzymes are primarily 3–10 bp. The corresponding electropherogram showed a clear smear of DNA degradation fragments in the 300–2000 bp range for the DNase I group, whereas no signal was visible in the PIL‐P‐Ce sample (Figure 2I). This evidence supports that PIL‐P‐Ce can effectively combine both photocatalytic and nuclease‐mimetic activities for rapid and efficient cleavage of bacterial DNA.

Several possible mechanisms exist for metal ions to hydrolyze phosphodiester bonds.^[^ ^24^ ^]^ To explore the theoretical mechanism of DNA hydrolysis by PIL‐P‐Ce, quantum chemistry calculations were used to construct and optimize different reaction complex (RC) models for Ce^4+^ ions and DNA analogues.^[^ ^25^ ^]^ Bond formation, bond breaking, and energy barriers during the hydrolysis from the transition state (TS) to product complex (PC) were obtained. When Ce^4+^ ions undergo intramolecular nucleophilic activation, the product formation energies for two attack modes are significantly positive (46.11 & 34.05 kcal mol^−1^), suggesting that both endothermic reactions are unlikely to occur (Figure S9, Supporting Information). However, the binding of water/hydroxide molecules to Ce^4+^ ions assists either the leaving group (Figure 2J,K; Movie S1, Supporting Information) or the nucleophilic attack (Figure 2L,M; Movie S2, Supporting Information). This assists the P─O cleavage activation of phosphodiesters in DNA analogues with relatively small energy barriers (1.89 & 7.17 kcal mol^−1^), significantly lower than previously reported values (44.3 kcal mol^−1^).^[^ ^26^ ^]^ Moreover, the relative energies of PC are negative (−2.50 and −31.88 kcal mol^−1^), revealing that both reactions are exothermic and thus more likely to occur. These results indicate that PIL‐P‐Ce primarily operates via an indirect mechanism, where external assistance to Ce^4+^ ions facilitates efficient nuclease mimicry.

### 2.3 Effects on Bacterial and Their ARGs

**Figure 3:**
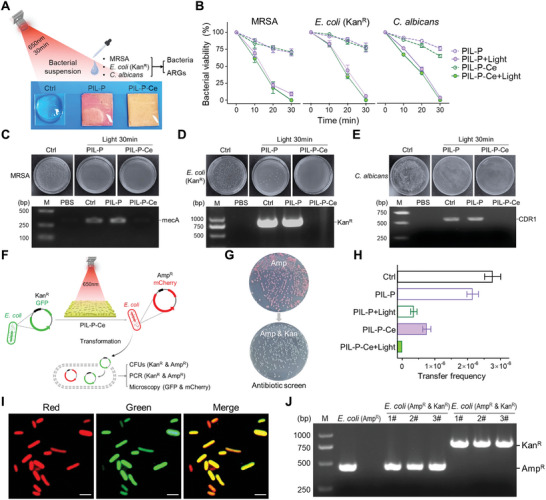
Effects on bacteria and their ARGs. A) Image showing bacterial suspensions of MRSA, *E. coli* (Kan^R^), and *C. albicans* drop‐casted onto the control PET and test PIL‐P‐based membranes, and then subjected to 650 nm light for 30 min. The survival of bacteria and ARG content were evaluated. B) Bacterial viabilities of three strains in PIL‐P‐based groups with or without light for 30 min (n = 3). C–E) Colony counts and PCR analyses for ARGs of the bacterial strains after light treatment. F) Schematic of an experiment testing PIL‐P‐Ce's role in preventing ARG transmission. Plasmids from *E. coli* BL21 (carrying GFP and Kan^R^ genes) were incubated with the membranes, and then mixed with *E. coli* DH5α (plasmid carrying mCherry and Amp^R^ genes) for transformation. Colonies screened by dual antibiotics were counted to evaluate ARG transfer efficiency, verified by PCR and fluorescence analyses. G) Colony photos of *E. coli* DH5α pre and post transformation. H) Transfer frequency of plasmid ARGs (n = 3). I) Fluorescence images of *E. coli* colonies screened with two antibiotics, all showing dual‐color fluorescence. Scale bar, 2 µm. J) Agarose gel PCR detection of screened *E. coli* (Kan^R^ & Amp^R^).

PIL‐P‐Ce membranes were tested against three drug‐resistant microorganisms: MRSA (mecA), *E. coli* (Kan^R^), and *C. albicans* (CDR1). After incubating with microbial suspensions with or without light for 30 min, the antibacterial performance of the membranes was evaluated via colony assay (**Figure** **3**A). All microbes decreased rapidly with light exposure, with almost no viable colonies in PIL‐P‐Ce group and ≈10% viability in PIL‐P group. Without light, however, 60–80% of microbes on PIL‐P membranes remained alive at 30 min, though nearly all were eliminated by 4 h (Figure 3B; Figures S10 and S11, Supporting Information). Both PIL‐P and PIL‐P‐Ce showed similar antimicrobial performance against three microbes, enhanced significantly by light. This suggests that antimicrobial processes of cationic polymers require relatively long contact time, involving electrostatic action and hydrophobic damage.^[^ ^27^ ^]^ However, PDT, relying on ROS from photosensitizers, accelerates microbial death.^[^ ^28^ ^]^ Notably, PIL‐P‐Ce's overall antimicrobial effect surpasses PIL‐P's, likely due to increased electrostatic interactions between metal cations and bacteria. In sum, PIL‐P‐Ce synergizes the interactions of cationic polymers and photodynamic effects to achieve rapid, high‐efficiency, broad‐spectrum antimicrobial performance.

PCR assays were used to quantify bacterial ARGs after 30 min of light exposure. While both membranes had comparable antibacterial efficiencies, they differed significantly in their impact on bacterial ARGs. Compared to the control, all three ARG bands vanished in PIL‐P‐Ce samples but remained unchanged in PIL‐P samples (Figure 3C–E). Notably, mecA and CDR1 genes exist in MRSA and *C. albicans* chromosomes, while Kan^R^ is in *E. coli* plasmids. This implies that after killing bacteria, PIL‐P‐Ce adsorbs the negative DNA of microbial chromosomes and plasmids via electrostatic action, followed by synergistic photocatalytic and nuclease‐mimetic activities to cleave ARGs.

Clinically, mixed infections, especially severe bacterial ones often coexist with fungal infections like *Candida*.^[^ ^29^ ^]^ Using three representative strains, we demonstrated PIL‐P‐Ce's potency against Gram‐positive, Gram‐negative bacteria, fungi, and its ability to degrade ARGs, offering a solution to control resistant microbial infections at the source.

To evaluate the PIL‐P‐Ce' role in preventing bacterial drug resistance spread, two *E. coli* strains with different ARGs and fluorescent genes were used for transformation experiments.^[^ ^30^ ^]^ Plasmid solution from BL21 (carrying green GFP and Kan^R^ genes) was incubated with the PIL‐P‐based membranes under 30 min of light, then mixed with DH5α (plasmid carrying red mCherry and Amp^R^ genes) for transformation, followed by antibiotic screening, PCR, and fluorescence assays (Figure 3F). Photos show that pristine DH5α can grow on the Amp plate, forming red colonies, while transformed bacteria could survive on the plate with both Amp and Kan, turning into white colonies after acquiring the BL21 strain's plasmid (Figure 3G; Figure S12, Supporting Information). Bacteria screened by dual antibiotics displayed both red and green fluorescence in all cells and showed product bands of Kan^R^ (816 bp) and Amp^R^ (449 bp) simultaneously in random samples (Figure 3I,J; Table S1, Supporting Information). This suggests that ARGs can be transferred, contributing to drug resistance spread among bacteria. Thus, it is crucial to degrade ARGs from dead bacteria.

Transfer frequency of ARGs in each test group was determined based on colony counts screened by dual antibiotics. Fewer colonies indicate lower frequency, implying more plasmid destruction and thus better prevention of drug resistance spread. Without light treatment, all groups exhibited transformed bacteria, with PIL‐P close to the control, with frequencies of 2.14 × 10^−6^ and 2.77 × 10^−6^, one order of magnitude higher than PIL‐P‐Ce (3.38 × 10^−7^). With light exposure, PIL‐P decreased by two‐thirds to 7.15 × 10^−7^, but no colonies were observed in PIL‐P‐Ce, demonstrating its superior DNA cleavage performance to block bacterial resistance spread (Figure 3H; Figure S12, Supporting Information).

### 2.4 Effects on Viruses and Their Nucleic Acids

**Figure 4:**
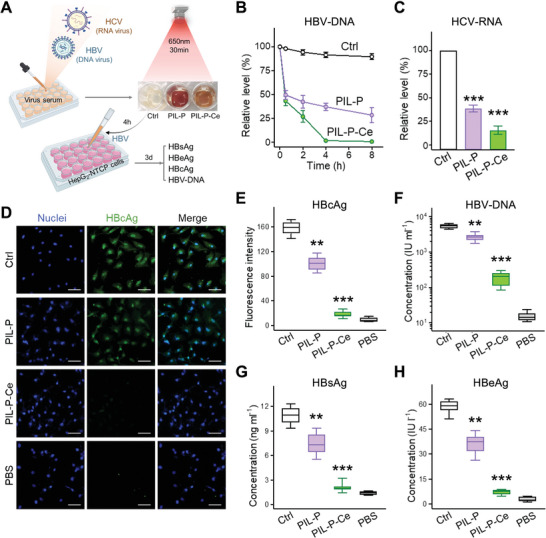
Effects on viruses and their nucleic acids. A) Flow chart outlining the membranes' role in testing viruses and their nucleic acids. Viral sera of HBV and HCV were dropped onto the control PET and tested PIL‐P‐based membranes, followed by 30 min light exposure and further incubation without light. Virus load was assessed by PCR assays for viral nucleic acid. After 4 h contact with the test membranes, HBV samples were introduced into HepG2‐NTCP cells to evaluate the infectivity of residual viruses. HBV antigen levels (HBsAg, HbeAg, and HBcAg) and HBV‐DNA in the hepatocyte samples were determined at 3 day‐post‐infection. B) Line chart showing the timeline of HBV‐DNA reduction across groups. C) HCV‐RNA level after 4 h contact with the test membrane. D,E) Immunofluorescence assays for HBcAg expression in HBV‐infected hepatocytes per test group. Scale bar, 50 µm. F–H) HBV‐DNA and HBV antigen (HbsAg and HbeAg) levels in hepatocyte culture mediums for each group. PBS was used as a negative control. N  =  3. ^**^ *p* < 0.01, ^***^ *p* < 0.001, PIL‐P‐based membranes are compared to PET control and analyzed by Student's *t*‐test.

To investigate the efficacy of PIL‐P‐Ce against two types of viruses, HBV and HCV. Viral sera from patients were incubated with the test membranes with or without light for 30 min. The viral load was then quantified using qPCR assays for HBV‐DNA or HCV‐RNA levels. After 4 h of contact with the membranes, HBV samples were added to human HepG2‐NTCP cells to assess the infectivity of residual virus, by determining the levels of HBV antigens (HBsAg, HBeAg, and HBcAg) and HBV‐DNA at 3 days post‐infection (**Figure** **4**A). With 30 min of light, the PIL‐P‐based membranes' effectiveness against viruses was not as strong as against bacteria, reducing HBV‐DNA by ≈60% and 50% in PIL‐P‐Ce and PIL‐P groups, respectively. After 4 h of contact, PIL‐P‐Ce inactivated ≈98% of HBV‐DNA, whereas 40% remained in PIL‐P (Figure 4B). This suggests that viral nucleic acids are harder to destroy than bacterial ones, as they are protected by both protein capsids and lipid envelopes. Under the same conditions, HCV‐RNA levels dropped ≈85% and 65% in PIL‐P‐Ce and PIL‐P, respectively (Figure 4C). These results suggest that PIL‐P‐Ce can effectively inactivate both DNA and RNA viruses, but require longer contact time than for its antibacterial activity. The slightly lesser efficacy against HCV‐RNA might be due to the hydroxyl group on RNA ribose, which could increase steric hindrance for the breakage of phosphodiester bonds.^[^ ^31^ ^]^

Viral nucleic acid is the benchmark for assessing viral replication, with direct implications on disease infectivity, progression, and prognosis.^[^ ^5^, ^32^, ^33^ ^]^ Besides HBV and HCV, common clinical DNA viruses include human papillomavirus (HPV) and Epstein–Barrvirus (EBV), while RNA viruses encompass human immunodeficiency virus (HIV) and SARS‐CoV‐2. Therefore, our PIL‐P‐Ce holds the potential to eliminate infectious nucleic acids from a broader spectrum of viruses, enabling control of infections at their source.

The antiviral effect of PIL‐P‐Ce was further verified by observing hepatocytes infected by residual HBV after treatment. Cellular HBcAg fluorescence in PIL‐P was as visible as PET control, but hardly detectable in PIL‐P‐Ce, similar to a PBS negative control (Figure 4D,E). The viral antigens and DNA in the hepatocellular supernatant were examined for each group. HBV‐DNA in PIL‐P‐Ce decreased by 96%, along with reductions in HBsAg and HBeAg levels by 81% and 88%, respectively; the three HBV indicators in PIL‐P decreased by less than half (Figure 4F–H) Without the initial 30 min of light, PIL‐P‐Ce reduced HBV‐DNA by only ≈30% even with 8 h of contact, and the residual virus could still infect liver cells. The antiviral efficacy of PIL‐P was much worse, with only half of that of PIL‐P‐Ce (Figure S13, Supporting Information). Overall, these results confirmed that PIL‐P‐Ce could effectively eliminate viruses under light conditions, thereby preventing virus transmission.

### 2.5 Wound Therapeutic Test on MRSA‐HBV Co‐Infected Mice

**Figure 5:**
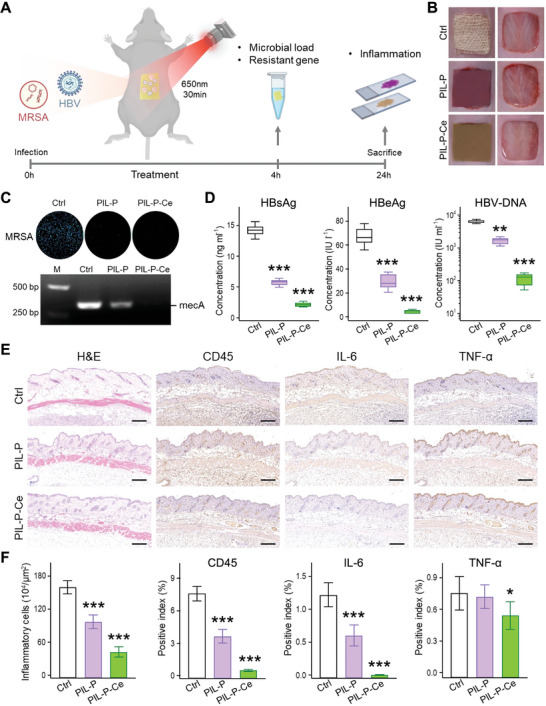
Wound therapeutic efficacy on MRSA‐HBV co‐infected mice. A) Flow chart illustrating wound treatment evaluation of PIL‐P‐based membranes in a mixed infected mouse model. Skin wounds were inoculated with a mixture of MRSA and HBV and covered with tested membranes with 650 nm light for 30 min. Sterile gauze was used as control. After 4 h, dressings were removed and soaked in sterile PBS solutions for further pathogen, ARG, and nucleic acid analysis. At 24 h post infection, mice were sacrificed, and infected tissues were excised for inflammation assessment. B) Photos of skin wounds at 4 h post treatment. C,D) Quantification of bioluminescent MRSA colonies, mecA ARGs, HBV antigens (HBsAg and HBeAg), and HBV‐DNA in dressing soaking solutions. E,F) Histological analyses of infected skins at 24 h by H&E and immunohistochemical staining for inflammatory cells and CD45, TNF‐α, and IL‐6 expressions. Scale bar, 100 µm. N  =  3. ^*^ *p* < 0.05, ^**^ *p* < 0.01, ^***^ *p* < 0.001, PIL‐P‐based membranes are compared to control and analyzed by Student's *t*‐test.

The in vivo performance of PIL‐P‐Ce was evaluated using a mouse skin wound model co‐infected with MRSA and HBV. The wounds were inoculated with a microbial mixture, and then treated with PIL‐P‐based membranes under light for 30 min. After 4 h, the membranes were removed from wounds and soaked in PBS solution for analyzing microbial load and resistance gene analysis. Wound tissues were further examined for inflammation 24 h post infection (**Figure** **5**A).

Compared to the control, both PIL‐P and PIL‐P‐Ce treated wounds had no visible changes or bacterial colonies, indicating effective MRSA killing. Concurrently, PCR assay for mecA levels revealed absence of this resistance gene in PIL‐P‐Ce, unlike in both PIL and control groups (Figure 5B,C; Figure S14, Supporting Information). Moreover, PIL‐P‐Ce reduced HBsAg by 85% (from 14 to 2 ng mL^−1^), HBeAg by 93% (from 67 to 4 IU L^−1^), and HBV‐DNA by 98% (from 6300 to 120 IU mL^−1^). In comparison, PIL‐P only managed ≈60% reduction, with ≈2000 IU mL^−1^ of HBV‐DNA remained (Figure 5D). As HBV‐DNA above 1000 copies mL^−1^ (≈180 IU mL^−1^) indicates HBV infectivity, PIL‐P‐Ce not only inactivated viruses but also controlled their transmission. These results align with its in vitro efficacy against bacteria and viruses (Figures 3 and 4).

Histological and immunohistochemical tests were conducted on wound skin tissues at 24 h. Leukocyte antigen CD45 labels infiltrating inflammatory cells, with IL‐6 and TNF‐α indicating concurrent inflammation. In the control, high amounts of these markers indicate active infection‐induced inflammation. In contrast, CD45^+^ cells and IL‐6 signals in PIL‐P‐Ce group were almost entirely reduced, and TNF‐α also decreased. Notably, PIL‐P's reduction of CD45 and IL‐6 signals was about half of that in PIL‐P‐Ce, paralleling the HBV response for each group (Figure 5E,F). This implies that viral infections can also induce inflammation like bacteria. The in vivo results demonstrate that PIL‐P‐Ce possesses robust antimicrobial and antiviral properties, coupled with ARG and viral nucleic acid cleavage abilities, thereby effectively controlling inflammation and curbing infection from the source.

### 2.6 In Vivo Phototoxicity Assessment by MS‐Based Proteomics

**Figure 6:**
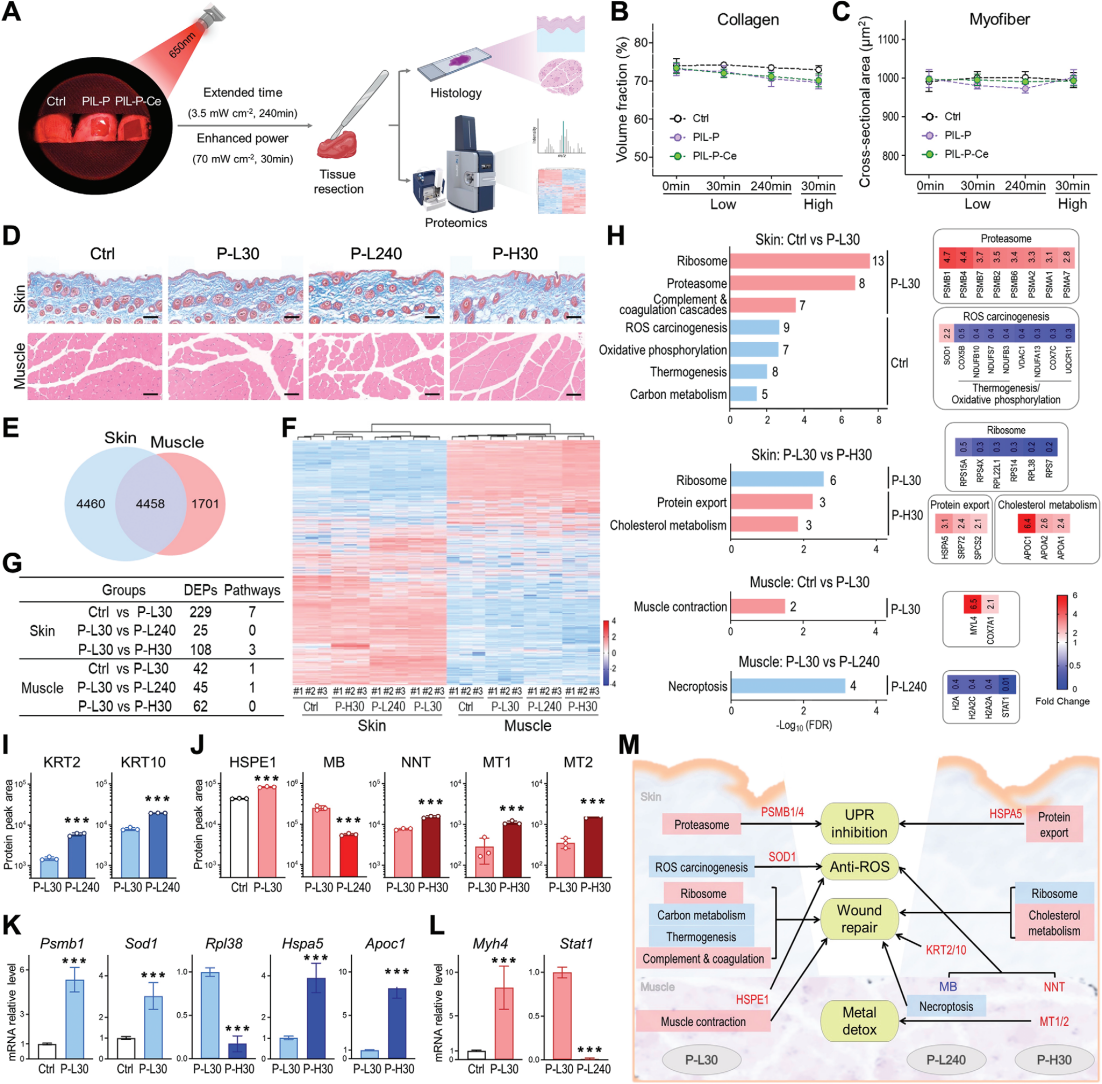
Proteome‐wide assessment of reducing phototoxicity to normal tissues. A) Schematic of in vivo phototoxicity evaluation for PIL‐P‐based membranes. Mouse back wounds were covered with tested membranes and subjected to two light conditions: extended time (3.5 mW cm^−2^, 4 h) and enhanced power (70 mW cm^−2^, 30 min). Sterile gauze acted as control. Skin and muscle tissues from wound sites underwent for histological and MS‐based proteomic studies. B,C) Quantification of skin collagen volume and myofiber cross‐sectional area based on Masson and H&E staining, respectively. D) Histological images of skin (Masson staining) and muscle (H&E staining) for PIL‐P‐Ce groups. Sterile gauze under treatment condition (3.5 mW cm^−2^, 30 min) served as control. Scale bar, 100 µm. E) Venn diagram depicting proteins quantified in skin (blue) and muscle (red). F) Heatmap of tissue proteomic data. G) of DEP counts and enriched pathways in compared groups. H) KEGG‐enriched pathways (left) and associated DEP fold changes (right). Each pathway's DEP count is labeled on the bar's right. I,J) MS‐quantified peak areas of major DEPs outside KEGG pathways in skin and muscle. K,L) mRNA levels of representative DEPs from KEGG pathways in skin and muscle, determined by qPCR. M) Wound tissue proteomic responses to PIL‐P‐Ce under varying light conditions. Red indicates activated pathways/DEPs, and blue indicates inhibited ones. N  =  3. ^***^ *p* < 0.001, analyzed by Student's *t*‐test.

To further assess the in vivo phototoxicity of PIL‐P‐based membranes, the mouse wound model without microbial infection was exposed to two light modes: extended time (3.5 mW cm^−2^, 4 h) and enhanced power (70 mW cm^−2^, 30 min). Both were compared to the standard treatment (3.5 mW cm^−2^, 30 min). Skin and muscle tissues from the wound area were collected for histological and proteomic analyses (**Figure** **6**A).

Histological changes of wounds were observed via Masson and H&E staining, and tissue injury was assessed by quantifying skin collagens and muscle fibers. Minimal differences in tissue structure, cell morphology, skin collagens and muscle fiber were found between PIL‐P‐based groups and the control, regardless of light modes (Figure 6B–D; Figure S15, Supporting Information). This indicates that the PIL‐P‐based membranes' impact on wound tissue morphology is negligible, regardless of light exposure duration or intensity.

High‐throughput MS‐based quantitative proteomic analyses were performed on skin and muscle samples from PIL‐P‐Ce groups to explore tissue response at the molecular level. A total of 10080 skin proteins and 7192 muscle proteins were identified. Among these, 8918 in skin and 6159 in muscle were quantified, with 4458 proteins common to both tissues (Figure 6E). A clear distinction between the proteomes of two tissues was evident, skins had more highly‐expressed proteins in heatmap and a more diverse distribution in PCA plot than muscles (Figure 6F; Figure S16, Supporting Information), potentially due to the skin's complex tissue structure.

For each tissue type, the proteome of PIL‐P‐Ce under the treatment condition (P‐L30) was first compared to the corresponding control. Subsequently, P‐L30 was compared to groups receiving extended light exposure (P‐L240) or enhanced light power (P‐H30), to assess phototoxic effects on the tissue proteome (Figures S17 and S18, Supporting Information).

In skin, there were 120 upregulated and 109 downregulated differentially expressed proteins (DEPs) when comparing P‐L30 samples to controls (Figure 6G; Figure S19A, Supporting Information). Pathway analyses revealed that ribosome, proteasome, complement and coagulation cascades were upregulated, while ROS chemical carcinogenesis, thermogenesis, oxidative phosphorylation, and carbon metabolism were downregulated (Figure 6H; Figures S20 and S21, Supporting Information). ROS carcinogenesis pathway involves eight down‐regulated DEPs, with seven also participating in thermogenesis and oxidative phosphorylation, however, superoxide dismutase SOD1, a key skin antioxidant, increased. Moreover, the rise in proteasome subunits PSMB1/4 helps remove proteins misfolded by ROS damage. Therefore, under treatment, PIL‐P‐Ce facilitates early wound repair and hemostasis, adapts to the environment via energy reduction, and resists ROS oxidative damage and unfolded protein response (UPR).^[^ ^34^, ^35^ ^]^ When the light exposure time was extended eight‐fold, the proteome heatmap of P‐L240 was similar to that of P‐L30, and PCA plot showed both groups approached a positive value of 10 on PC1 axis (Figure S17, Supporting Information). No enriched KEGG pathways between P‐L240 and P‐L30 groups, yet 25 DEPs, including elevated keratins (KRT2/10) were identified (Figure 6G,I; Figure S19B, Supporting Information). This implies activated keratinocytes facilitated wound repair,^[^ ^36^ ^]^ indicating skin's tolerance to prolonged, low‐dose light exposure. Upon a 20‐fold increase in light power for the same 30 min exposure, 108 DEPs were observed between P‐H30 and P‐L30 groups, and one downregulated ribosome pathway and two upregulated protein export and cholesterol metabolism pathways were enriched (Figure 6G and H; Figures S19C and S22, Supporting Information). Despite the increase of heat shock protein (HSPA5) in the protein export pathway, which had some inhibitory effect on PDT‐induced UPR,^[^ ^37^ ^]^ it could not counteract ROS overload, leading to inhibited protein synthesis and increased catabolism.

In muscle, 42 DEPs were identified between P‐L30 and control samples, along with one pathway related to muscle contraction (Figure 6G,H; Figures S23A and S24A, Supporting Information), indicating that PIL‐P‐Ce treatment could potentially reduce the wound size by maintaining local muscle tension. Moreover, elevated heat shock protein (HSPE1) helps prevent PDT‐associated misfolded protein production (Figure 6J). Although the proteomic profiles were similar with differing light exposure times, the P‐L240 group identified 45 DEPs, along with a downregulated necroptosis pathway (Figure 6G,H; Figure S23B and S24B, Supporting Information). Given that necroptosis has been linked with PDT,^[^ ^38^ ^]^ and that necroptosis in muscle fibers can stimulate the proliferation of muscle stem cells,^[^ ^39^ ^]^ this suggests that when the phototherapy is prolonged, PIL‐P‐Ce might affect tissue regeneration through modulating muscular necroptosis. Additionally, reduced myoglobin (Mb) could affect ROS production by limiting local oxygen supply, thereby mitigating tissue damage (Figure 6J; Figure S23B, Supporting Information). With light power up 20‐fold, 62 DEPs were found in the P‐H30 muscle group, with no pathway enriched. Notably, nicotinamide nucleotide transhydrogenase (NNT) and metallothionein (MT1/2) increased significantly, reducing ROS via mitochondria^[^ ^40^ ^]^ and detoxifying material by binding to Ce ions,^[^ ^41^, ^42^ ^]^ respectively (Figure 6G,J; Figure S23C, Supporting Information).

To validate the proteomic findings, MS‐based protein quantification and qPCR analyses were used to compare representative DEPs and genes expressed in different groups (Figure 6I–L; Figures S21,S22,S24, Supporting Information). As expected, changes at both protein and gene levels from the enriched pathways were consistent with the proteomic results.

In summary, the in vivo phototoxicity results indicate that both skin and muscle tissues appear morphologically unaffected by the phototoxic effects of the materials. However, high‐throughput proteomic analysis can unveil molecular‐level changes and mechanisms under varying conditions.^[^ ^43^ ^]^ As an internal tissue, muscles can be prone to proteomic abnormalities if the skin's protective barrier is lost, even with longer exposure to low‐dose light. However, the impact of enhanced light power on muscles seems to be limited, probably due to their rich blood supply, which facilitates the timely removal of excess ROS (Figure 6M). Notably, the proteomic responses in different tissues reflect their unique anatomical locations and compositions, which cannot be obtained from in vitro cell culture. Under phototherapeutic conditions, PIL‐P‐Ce enhances wound repair. Skin exhibits greater tolerance to prolonged light exposure from PIL‐P‐Ce, while muscle can endure higher light intensity. Singlet oxygen generated by porphyrins has an extremely short lifespan (3–4 µs) and diffusion distance (≈50 nm), thereby confining photodynamic effect to the irradiation site and enhancing biosafety.^[^ ^14^ ^]^ The biocompatibility of cerium ions has been well established. Through both in vitro and in vivo tests, this study confirms that PIL‐P‐Ce membranes present minimal phototoxicity, highlighting their excellent biosafety.

## 3 Conclusion

Ce(IV)‐complexed imidazolium‐based poly(ionic liquid)/porphyrin nanofibrous membrane, PIL‐P‐Ce, was prepared. Its potent, stable, and broad‐spectrum antimicrobial and antiviral performances, along with ARGs and viral DNA/RNA cleavage abilities under light were investigated. The underlying nuclease‐mimetic and photocatalytic mechanisms of PIL‐P‐Ce were elucidated. Tests on mouse skin wounds co‐infected with MRSA and HBV demonstrated PIL‐P‐Ce's potential as a “green” antimicrobial and antiviral dressing with inherent nuclease activity, capable of hindering the spread of infections. Proteomic analysis confirmed the material's minimal tissue phototoxicity, enhancing its prospects for clinical application.

## 4 Experimental Section

### Materials

*Chemical reagents*: 1‐Vinylimidazole, 1‐bromohexane, bromoacetic acid, n‐carbobenzoxy‐L‐lysine (CBZ‐LYS), palladium‐carbon (Pd/C), acrylonitrile, azobis‐(isobutyronitrile) (AIBN), N,N‐dimethylformamide (DMF), toluene and polyacrylonitrile (PAN) (Mw:150000) were purchased from Shanghai Chemical Reagents Co. Dowex 50WX8 resin, TCPP, Ce(NH_4_)_2_(NO_3_)_6_, BNPP, p‐nitrophenol, 9,10‐anthracenediyl‐bis(methylene) dimalonic acid (ABDA), formic acid, acetonitrile and Triton X‐100 were purchased from Shanghai Aladdin Biochemical Technology. All chemicals were analytical grade, and deionized water was consistently used.

### Microorganisms and Cell Lines

MRSA (ATCC 33 591), *C. albicans* (ATCC 76 615) were provided by Dr. Shengwen Shao (Huzhou University School of Medicine, China). Ampicillin‐resistant *E. coli* DH5α (pKK233‐2 plasmid: mCherry gene, Amp^R^) and kanamycin‐resistant *E. coli* BL21 (pET28a plasmid: GFP gene, Kan^R^) were gifted from Dr. Yanran Chen (Shanghai Institute of Biochemistry and Cell Biology, CAS). Serum samples with HBV or HCV came from chronic hepatitis patients. Human hepatoma cell HepG2‐NTCP, sensitive to HBV due to overexpression of sodium taurocholate cotransporting polypeptide (NTCP: a receptor for HBV), was from Liver Cancer Institute, Zhongshan Hospital of Fudan University. Human dermal fibroblasts (HDF) and human embryonic kidney 293T cells (HEK293T) were from Shanghai Ninth People's Hospital of China.

### Biological Reagents

Luria‐Bertani broth medium (LB), Yeast Extract Peptone Dextrose medium (YPD), kanamycin (Kan), ampicillin (Amp), DNase I, and EDTA were from Biosharp. Lycopene (HY‐N0287) was from MedChemExpress; DNA polymerase (KFX‐101) and SYBR qPCR Mix (QPS‐201) from TOYOBO; reverse transcription reagent kit (RR036A) from Takara. DNA primers were made by Tsingke Biotech. Cell culture reagents were from Sigma–Aldrich. BCA protein assay (P0009), MTT assay (C0009S), H&E staining (C0105M), and RNA extraction (R0026) kits were from Beyotime Biotech. DNA extraction kits for cellular (D3122‐02), bacterial (D3146‐02), and plasmid (P1114‐02) were from Magen Biotech. 8‐oxoguanine ELISA kit (CB11596‐Hu) was from Coibo Biotec. ELISA Kits for HBsAg (JL13634) and HBeAg (JL20009) were from Jianglai Biotech. HBV‐DNA (S3118E) and HCV‐RNA (S3119E) quantification kits were from Sansure Biotech. HBcAg antibody (MA1‐7607) was from Thermo Fisher. Mouse IgG antibody conjugated with Alexa Fluor 488 (ab150105), DAPI (ab104139), CD45 antibody (ab10558), IL‐6 antibody (ab208113), and TNF‐α antibody (ab1793) were from Abcam. Rabbit IgG antibody (111‐035‐045) was from Jackson Immuno Research. Donkey serum (SL050) was from Solarbio Science & Tech.

### Characterization

^1^H NMR spectra of the synthesized substances were analyzed on a Varian 400 MHz spectrometer using d_6_‐DMSO and D_2_O as solvents. FT‐IR spectra of the membranes were taken on a Nicolet 5200 spectrometer, ranging from 600 to 4000 cm^−1^. XPS profiles were measured on a Rigaku XPS‐7000 spectrometer with a Mg Kα X‐ray source. Membrane morphologies were assessed on a Hitachi S‐4700 field emission SEM equipped with energy‐dispersive detector. The diameters of the nanofibers were examined using Image‐Pro Plus software. TEM and element mapping measurements were detected by a FEI Tecnai F20 microscope. The content of Ce ions was determined using Thermo Scientific iCAP RQ ICP‐MS. Static contact angle was examined by a Krűss DSA10 Drop Shape Analyzer. TGA was performed on PerkinElmer TGA 4000, with temperature between 30 and 800 °C under N_2_, and a heating rate of 20 °C min^−1^. The photocatalytic activity of the materials was assessed on a Ceaulight CEL‐S350 xenon light source system with a 650 nm optical filter. Both low (3.5 mW cm^−2^) and high (70 mW cm^−2^) power settings were used for treatment and phototoxicity evaluation. Optical density (OD) and UV–vis values were measured with a BioTek Epoch2 spectrophotometer. The characterization methods for PIL‐P‐based membranes' physical properties are as follows:

### Air Permeability

A 2 cm^2^ membrane was placed over a 50 mL water‐filled bottle (initial weight: M_0h_) and kept at 37 °C with 75% humidity for 24 h (final weight: *M*_24h_). The control (*M*_control_) lacked a membrane. The permeability rate was calculated as following equation:

(1) $$\mathrm{Permeability}\;\mathrm{rate}\;\left(\%\right)=\frac{M_{24h}-M_{0h}}{M_{\mathrm{control}}-M_{0h}}\times 100\%$$

### Water Uptake

A membrane was dried overnight at 55 °C (weight: *M*_0h_), then submerged in 10 mL water for 24 h and reweighed (*M*_24h_). The uptake rate was computed as following equation:

(2) $$\mathrm{Water}\;\mathrm{uptake}\;\mathrm{rate}\;\left(\%\right)=\frac{M_{24h}-M_{0h}}{M_{0h}}\times 100\%$$

### Water Swelling

Membrane thickness was measured before (*T*_dry_) and after (*T*_wet_) water soaking. Water swelling rate was calculated as following equation:

(3) $$\mathrm{Water}\;\mathrm{swelling}\;\mathrm{rate}\;\left(\%\right)=\frac{T_{\mathrm{wet}}-T_{\mathrm{dry}}}{T_{\mathrm{dry}}}\times 100\%$$

### Protein Adsorption

A membrane (1 cm^2^) was soaked in 1 mL of 5 wt.% BSA in PBS at 37 °C for 2 h, followed by three PBS rinses. Adsorbed proteins were sonicated off the membrane at 40 KHz in 1 mL of 1 wt.% SDS in PBS, then quantified with a Micro BCA assay.

### Fabrication of PIL‐P‐Ce Nanofibrous Membranes

Initially, two monomers, the ionic liquid 3‐hexyl‐1‐vinylimidazolium bromide ([HVIm][Br]) and metal ligand 2,2′‐(5‐acrylamido‐1‐carboxypentylazanediyl) diacetic acid (AANTA), were synthesized.^[^ ^10^ ^]^ Their copolymer, P(IL‐co‐AANTA‐co‐AA), was prepared via free radical copolymerization as described previously.^[^ ^10^ ^]^ Next, a mixture containing P(IL‐co‐AANTA‐co‐AA) (0.85 g), TCPP (0.0285 g), and PAN (0.26 g) were dissolved in 8 mL of DMF and stirred overnight to obtain the precursor solution for electrospinning. PIL‐P nanofibrous membranes were fabricated using a QZNT‐E01 electrospinning machine (Foshan Lepton Precision M&C Tech) at a 14 kV and a flow rate of 1 mL h^−1^, then vacuum‐dried. Lastly, PIL‐P‐Ce were obtained by immersing PIL‐P membranes (3 cm^2^) in a Ce(NH_4_)_2_(NO_3_)_6_ solution (60 mL, 0.009 m) at room temperature for 2 h. The obtained PIL‐P‐Ce membranes were ultrasonically washed thrice to remove adsorbed Ce^4+^ ions and vacuum‐dried under for use.

### Singlet Oxygen Measurement

ABDA was used to indicate singlet oxygen (^1^O_2_) production from the membranes during light irradiation.^[^ ^15^, ^16^ ^] 1^O_2_ specifically degrade ABDA, reducing its absorbance at 378 nm (A_378_). PIL‐P‐based membranes (1 cm^2^) were soaked in 1 mL of a ddH_2_O/DMSO (99:1 v/v) solution with ABDA (100 µm) and irradiated under 650 nm light (3.5 mW cm^−2^). A_378_ of the solution was measured (*A*) over time. An ABDA solution without a membrane used as a blank, while one with TCPP (100 µm) served as a positive control. The degradation ratio (*A/A_0_*) of ABDA gauged ^1^O_2_ production efficiency from the PIL‐P‐based membranes during light exposure, with *A_0_* as the initial absorbance.

### Cytotoxicity and Hemolysis Assessment

HDF and HepG2‐NTCP cell lines were employed to assess the toxicity of PIL‐P‐based membranes by MTT assay. Cells were cultured in Dulbecco's Modified Eagle Medium (DMEM) containing 10% fetal bovine serum. After a 72 h culture in a 24‐well plate with cell inoculation (≈3 × 10^4^ per well) in 1 mL medium, sterilized membranes (1 cm^2^) were added to each well, and exposed to light for the first 30 min or not. Cell viability was evaluated 24 h later by absorbance at 490 nm, and cell morphology was observed with an Olympus IX71 microscope. The relative growth rate (RGR) was calculated as following equation, with PET as a control:

(4) $$\mathrm{RGR}\;\left(\%\right)\;=\frac{OD_{\mathrm{sample}}}{OD_{\mathrm{control}}}\times 100\%$$

Fresh human red blood cells (RBCs) from volunteers were diluted to 2% in PBS for hemolysis assay. Sterilized membranes (1 cm^2^) were added to 2 mL of this RBC. After 3 h, with or without a 30 min light exposure, the optical density at 576 nm was recorded to evaluate hemoglobin release. RBC in 2% Triton and PBS served as positive and negative controls, respectively. Hemolysis rate was calculated as following equation:

(5) $$\mathrm{Hemolysis}\;\mathrm{rate}\;\left(\%\right)=\frac{OD_{\mathrm{sample}}-OD_{\mathrm{negative}\;\mathrm{control}}}{OD_{\mathrm{positive}\;\mathrm{control}}-OD_{\mathrm{negative}\;\mathrm{control}}}\times 100\%$$

### BNPP Degradability

PIL‐P‐based membranes (1 cm^2^) were immersed in 5 mL BNPP (pH 7.8, 50 mm in Tris‐HCl buffer) for 4 h with or without 30 min light exposure. At intervals, 300 µL samples were diluted to 3 mL and measured using UV–vis spectrometry at 400 nm (A_400_). A_400_ values indicate p‐nitrophenol production, assessing BNPP degradation. To evaluate PIL‐P‐Ce reusability, the tested membrane was rinsed twice with ddH_2_O and re‐tested. To determine if PIL‐P‐Ce‐mediated BNPP cleavage follows enzymatic kinetics, varying BNPP concentrations were incubated with membranes for 10 min and A_400_ measured. Using the p‐nitrophenol calibration curve, initial PIL‐P‐Ce cleavage rates were identified. The Michaelis constant (*K*_m_) was calculated using the Lineweaver‐Burk equation:

(6) $$\frac{1}{v}=\frac{K_{m}}{V_{max}\left[S\right]}+\frac{1}{V_{max}}$$

*V*_max_ is maximal velocity, *v* is initial velocity, and [*S*] is substrate concentration.

### DNA Decomposability

Bacterial genomic DNA and plasmid were extracted from *E. coli* (Kan^R^) using respective kits. To assess PIL‐P‐Ce's DNA decomposability, 500 µL solutions containing genomic DNA or plasmid (200 ng µL^−1^) were incubated with membranes (1 cm^2^) at 37 °C with or without 30 min light. DNA amounts were quantified on a Thermo Scientific NanoDrop 2000 spectrophotometer, and visualized through 1.5% agarose gel electrophoresis.

The mechanism behind PIL‐P‐Ce's nuclease‐like function was explored by studying various factors, including temperature (4–90 °C), pH (4.5–10.5), physiological ions such as Na^+^ (0–200 mm NaCl), Mg^2+^ (0–20 mm MgCl_2_) and PO_4_^3−^ (0–100 mm PBS), ^1^O_2_ scavenger (0–50 mm lycopene) and metal chelator (0–50 mm EDTA), on its DNA cleavage activity.

To compare PIL‐P‐Ce's DNA cleavage efficiency with DNase I, high resolution mass spectrometry (MS), along with agarose gel electrophoresis, was used to analyze DNA degradation products. 500 µL plasmid DNA (200 ng µL^−1^) was treated with PIL‐P‐Ce (1 cm^2^, under 650 nm light, 3.5 mW cm^−2^) or DNase I (100 U mL^−1^, the ratio of enzyme to substrate is 1:100) at 37 °C for 30 min. The products were analyzed on a nano Acquity UPLC system (Waters) coupled with a Q Exactive HF Orbitrap mass spectrometer (Thermo Scientific). Full MS scan in the m/z range was 250–2000 with a resolution of 30 000 full‐width half‐maximum (FWHM). Data were processed with Xcalibur 2.0.7 software (Thermo Scientific).

### 8‐Oxoguanine Detection

8‐Oxoguanine, a DNA damage product induced by ^1^O_2_, can generate from the photosensitizer component,^[^ ^26^ ^]^ porphyrin, in PIL‐P and PIL‐P‐Ce membranes upon light irradiation. To evaluate the photocatalytic activity of PIL‐P‐based membranes on DNA bases, human genomic DNA was extracted from HEK293T cells. DNA solutions (500 µL, 200 ng µL^−1^) were incubated with membranes (1 cm^2^) at 37 °C, with or without 30 min light exposure. 8‐oxoguanine production was quantified using an ELISA kit.

### DFT Calculations of DNA Hydrolysis Mechanisms

DNA hydrolysis by multinuclear metal complexes may involve base catalysis (via metal‐bound hydroxide) or acid catalysis (via metal ion or metal‐bound water). To decipher DNA hydrolysis mechanisms mediated by the Ce(IV) complex, various reaction complex (RC) models with two Ce^4+^ ions bound to a DNA analogue were constructed, and the related RC, transition state (TS), product complex (PC) were calculated based on quantum chemistry methods. During the computation, two direct modes involving intramolecular nucleophilic activation (Figure S9A–C, Supporting Information) with metal‐bound hydroxide (─OH) acting as a base to directly attack the DNA analogue, and two indirect modes either assisting the leaving group or catalyzing nucleophilic attack (Figure 2J–L) with metal‐bound water as acid catalysis were considered.

*The detailed simulations were summarized as follows*: All structures were fully optimized using density functional theory (DFT) with the B3LYP hybrid functional.^[^ ^44^, ^45^ ^]^ Mixed basis sets were used for structure optimization and frequency analysis: the effective core potential (ECP) basis set LANL2DZ was designated for the cesium atom, the splitting polarization basis set 6−31G (d, p) was used for C, H, O, N, and P atoms. Frequency analyses were utilized to distinguish possible transition states (one imaginary frequency) from local minimum states (zero imaginary frequency), and provided the zero‐point correction.

### Effects on Bacteria and Their ARGs

Drug‐resistant microorganisms, MRSA (mecA), *E. coli* (Kan^R^), and *C. albicans* (CDR1), were tested for antimicrobial properties of PIL‐P‐based membranes using the colony assay. Briefly, two bacteria and one fungus were cultured in LB and YPD media at 37 °C overnight. 200 µL microbial suspension (≈1 × 10^6^ CFUs) was added to the surface of sterilized membranes (1 cm^2^) and incubated with or without light for the first 30 min at 37 °C and high humidity (>90%). At intervals, 10 µL microbial suspension was streaked onto LB or YPD agar plates, and colony‐forming units (CFUs) were counted after 24 h. PET membranes were as controls. The antibacterial rate was calculated as following equation:

(7) $$\mathrm{Antibacterial}\;\mathrm{activities}\;\left(\%\right)=\frac{N_{\mathrm{control}}-N_{\mathrm{sample}}}{N_{\mathrm{control}}}\times 100\%$$

PCR evaluated the degradation effects of PIL‐P‐based membranes on ARGs. Simultaneously with the antimicrobial test, 1.5 µL microbial sample was amplified by PCR and visualized on a 1.5% agarose gel. Primers are in Table S1 (Supporting Information). The PCR mixture included KOD FX polymerase (0.5 µL), mixed primers (1.5 µL), 2x KOD FX buffer (12.5 µL), 2 mm dNTPs (5 µL), and ddH_2_O (4 µL), with amplification as previously reported.^[^ ^10^ ^]^

To study PIL‐P‐Ce's role against bacterial ARG spread, two *E. coli* strains, BL21 (with GFP and Kan^R^ genes) and DH5α (with mCherry and Amp^R^ genes), were used for transformation experiment.^[^ ^28^ ^]^ Briefly, 500 µL plasmid (200 ng µL^−1^) from BL21 were treated with PIL‐P‐based membranes (1 cm^2^) with or without 30 min light. The solution (1 µL) was mixed with 200 µL competent DH5α (pre‐treated with 0.05 m cooled CaCl_2_ for 30 min), After 30 min on ice, at 42 °C for 90 s, and 3 min on ice, 1 mL of antibiotic‐free LB liquid medium was added and incubated at 37 °C for 1 h. The mixture (100 µL) was then plated on LB agar with or without dual‐antibiotics (30 mg mL^−1^ Kan and 50 mg mL^−1^ Amp). Dual‐antibiotics screened colonies (*N*_transformant_) and unscreened colonies (*N*_competent_) were used to calculate the plasmid transfer frequencies as following equation, with transferred ARGs and fluorescent proteins verified through PCR and fluorescence analyses:

(8) $$\mathrm{Transfer}\;\mathrm{frequency}\;\left(\%\right)=\frac{N_{\mathrm{transformant}}}{N_{\mathrm{competent}}}\times 100\%$$

### Effects on Viruses and Their Nucleic Acids

To evaluate the antiviral efficacy of PIL‐P‐based membranes, HBV and HCV were tested using quantitative PCR (qPCR) and ELISA for nucleic acids and antigens, respectively. Viral sera (200 µL, ≈1 × 10^7^ IU mL^−1^) from patient were dropped onto membranes (1 cm^2^) and incubated at 37 °C, with or without light for 30 min. At intervals, 10 µL samples were analyzed for viral load via qPCR kits, quantifying HBV‐DNA and HCV‐RNA levels on Roche Lightcycler 480 analyzer.

To explore PIL‐P‐Ce's role in curbing viral transmission, HBV's infectivity to hepatocytes post‐treatment was tested. HepG2‐NTCP cells (≈6 × 10^4^ per well) were maintained in hepatocytes maintenance medium (PMM) with 4% PEG8000 in a 48‐well plate and mixed with 5 µL HBV serum after 4 h of contact with the tested membrane. A negative control used 5 µL PBS. Three days post‐infection, cell supernatants were collected, and HBV‐DNA and HBV antigens (HBeAg and HBsAg) secreted by infected cells were quantified via PCR and ELISA. Concurrently, intracellular HBcAg in HepG2‐NTCP cells was assessed through immunofluorescence. Cells were treated with HBcAg antibody (1:500) at 4 °C overnight, followed by Alexa Fluor 488‐conjugated anti‐mouse IgG (1:1000) at 37 °C for 45 min. After DAPI staining, fluorescence was observed under an Olympus BX53 microscope. Intensity was analyzed using Image‐Pro Plus 6.0 from three random views per sample.

### In Vivo Bacterial‐Viral Mixed Infection Treatment Study

Mice were handled according to protocols approved by Zhongshan Hospital's ethical committee. Healthy 6‐week‐old C57/B6 mice were housed under 12 h light‐dark cycles and anesthetized using pentobarbital sodium (50 mg kg^−1^). A dorsal wound (1 cm^2^) was created and inoculated with 100 µL bacterial‐viral suspension (containing ≈10^7^ CFUs MRSA and ≈10^6^ IU HBV‐DNA), then covered with tested membranes (1 cm^2^) under 650 nm light for 30 min. Sterile gauze served as control.

After 4 h, dressings were removed and soaked in 0.5 mL sterile PBS for 1 h. This dressing soaking solution was analyzed for microbial load and resistant gene. MRSA colonies were identified on agar plates and bioluminescent images captured using the MiniChemi 610 Plus system. ELISA and qPCR were used to assess HBV antigens and HBV‐DNA levels from the samples. MRSA colonies were counted from agar plates, while the mecA level was gauged using PCR. MRSA bioluminescent images were captured with a MiniChemi system (SinSage Tech). HBV antigens and DNA levels were assessed with ELISA and qPCR.

At 24 h post‐infection, mice were euthanized, and wound‐edge skin samples were obtained for hematoxylin‐eosin (H&E) and immunohistochemical (IHC) staining, following the manufacturer's instructions. Antibodies for IHC staining included: CD45 (1:200), IL‐6 (1:100), TNF‐α (1:100), and secondary anti‐Rabbit IgG (1:200). Images were captured using a Leica CS2 scanner. Three random fields were examined for each group, and brown granules indicated positive signals for CD45, IL‐6, and TNF‐α, analyzed using Image‐Pro Plus 6.0. The positive index was the ratio of positive to total cells.

### In Vivo Phototoxicity Study

To evaluate the potential phototoxicity of PIL‐P‐Ce on normal tissues, the mouse wound model was used. Non‐infected wounds (1 cm^2^) were covered with tested membranes (1 cm^2^). Two modes of light irritation, extended time (3.5 mW cm^−2^, 4 h) and enhanced power (70 mW cm^−2^, 30 min), were compared to treatment condition (3.5 mW cm^−2^, 30 min). Wound skin and muscle (≈5 mm thick) were sampled at intervals for Masson and H&E staining. Volume fraction of skin collagens and cross‐sectional area of muscle fibers were quantified using Image‐Pro Plus 6.0 software to evaluate tissue damage.

To systematically investigate PIL‐P‐Ce's phototoxicity, high‐performance liquid chromatography coupled with tandem mass spectrometry (HPLC‐MS/MS) was executed on tissue samples for proteomics. Samples were prepared as described previously.^[^ ^46^ ^]^ Briefly, tissues were lysed with protease inhibitors (Roche), sonicated, and centrifuged. Supernatants' protein content was measured using a BCA protein assay. 200 µg protein from each sample was reduced in dithiothreitol, alkylated with iodoacetamide, and then precipitated using precooled acetone. Protein samples underwent trypsin digestion (Promega, 1:50, 37 °C overnight), followed by desalting through SepPak C18 cartridges (Waters).

LC‐MS/MS analysis was performed using a nanoElute LC system coupled with TIMS‐TOF Pro mass spectrometry (Bruker). 200 ng peptides were loaded on an IonOpticks Aurora Series C18 column (25 cm × 75 µm, 1.6 mm), and eluted at a 200 nL min^−1^ flow rate (buffer A: 0.1% formic acid in water; buffer B: 0.1% formic acid in acetonitrile) with a 60 min gradient (0–45 min, 2% to 22% of buffer B; 45–55 min, 22% to 37% of buffer B; 55–60 min, 80% of buffer B). The mass spectrometry conditions included a scanning range of 100–1700 m/z in positive electrospray mode, an intensity threshold at 5000, and the accumulation and release time of 100 ms. A single cycle had a collection time of 1.16 s, which included one MS scan and ten parallel accumulation‐serial fragmentation (PASEF) secondary scans. MS data were processed using Peaks Online software (Bioinformatics Solutions, Inc.) against the SwissProt mouse database (17046 proteins, 2020.08.20 download). Parameters included trypsin digestion, fixed carbamidomethylation modification, variable modifications like acetylation, oxidation and deamidation, precursor mass error tolerance of 15 ppm, fragment mass error tolerance of 0.05 Da. Results of peptide‐spectrum matches (PSM) and proteins were filtered with a 1% false discovery rate (FDR). Proteins with over 50% missing values were excluded, and remaining blanks were filled with a random number between 0 and the minimum area value. The protein peak area was used for subsequent statistical analysis.

Differentially expressed proteins (DEPs) identified using a Wilcoxon rank‐sum test (FDR‐corrected *P*<0.01 and fold change (FC) >2 or <1/2). Principal component analysis (PCA) visualized proteomic profiles, and clustering analysis determined proteomic subtypes via the ConsensusClusterPlus R package (v.1.48.0).^[^ ^47^ ^]^ KEGG enrichment was conducted using the R package (v.4.1.1). The enriched terms and pathways with Benjamini‐Hochberg adjusted *P*<0.05 were considered significant, with an FDR cutoff of 0.05.

To validate proteomic findings, qPCR assays evaluated representative genes corresponding to DEPs in tissue samples. Total messenger RNA (mRNA) was isolated from tissues using RNAeasy kit. Reverse transcription was performed with PrimeScript Mix, and qPCR was done with SYBR Mix on a Bio‐Rad CFX96 system, following manufacturer's instructions. Relative gene expression was calculated using the ΔΔCT method, normalize to the *Gapdh* gene. Primers were in Table S2 (Supporting Information).

### Statistics and Reproducibility

All studies were performed independently at least three times. No statistical methods were used to predetermine sample sizes, but the sample sizes were based on previous studies.^[^ ^10^ ^]^ Animals were randomly assigned to treatment groups. No data were excluded from the analyses. All statistical analyses were performed using GraphPad Prism 9 software. Data are generally presented as mean ± standard deviation, unless otherwise indicated. For comparison between two groups, the two‐tailed, unpaired Student's *t*‐test was used. *p* < 0.05 was considered statistically significant, and additional indicators of statistical significance are provided accordingly in the text or in individual figure legends.

## Conflict of Interest

The authors declare no conflict of interest.

## Supporting information

Supporting Information

Supplemental Movie 1

Supplemental Movie 2

## Data Availability

The data that support the findings of this study are available from the corresponding author upon reasonable request.
