# Supplementary material for: Antimicrobial and Antiviral Nanofibers Halt Co‐Infection Spread via Nuclease‐Mimicry and Photocatalysis
Source: Adv Sci (Weinh). 2024 Apr 22;11(24):2309590. doi: 10.1002/advs.202309590 (PMC11200001; doi:10.1002/advs.202309590)
Supplement: Supplementary file 1 — Supporting Information [file ADVS-11-2309590-s002.pdf]

ADVANCED SCIENCE
Open Access

Supporting Information

for *Adv. Sci.*, DOI 10.1002/advs.202309590

Antimicrobial and Antiviral Nanofibers Halt Co-Infection Spread via Nuclease-Mimicry and Photocatalysis

Jieran Yao, Zhenhong Luo, Jiaying Lin, Na Meng, Jiangna Guo, Hui Xu, Rongwei Shi, Linhui Zhao, Jiateng Zhou, Feng Yan, Bin Wang* and Hailei Mao**

Supporting Information

Antimicrobial and Antiviral Nanofibers Halt Co-Infection Spread *via* Nuclease-Mimicry and Photocatalysis

Jieran Yao,[1] Zhenhong Luo,[2] Jiaying Lin,[1] Na Meng,[1] Jiangna Guo,[2] Hui Xu,[2] Rongwei Shi,[4] Linhui Zhao,[1] Jiateng Zhou,[3] Feng Yan,[2,] Bin Wang[3,]* and Hailei Mao[1,]**

[1]Prof. H. Mao, J. Yao, J. Lin, N, Meng, L. Zhao
Department of Critical Care Medicine
Zhongshan Hospital
Fudan University
Shanghai 200032, China.
E-mail: mao.hailei@zs-hospital.sh.cn

[2]Prof. F. Yan, Z. Luo, Dr. J. Guo, H. Xu
College of Chemistry
Chemical Engineering and Materials Science
Soochow University,
Suzhou 215123, China.
E-mail: fyan@suda.edu.cn

[3]Prof. B. Wang, J. Zhou
Department of Plastic and Reconstructive Surgery
Shanghai Ninth People's Hospital
Shanghai Jiao Tong University School of Medicine,
Shanghai 200011, China.
E-mail: wangb1435@sh9hospital.org.cn

[4]Dr. R. Shi
School of Material and Chemical Engineering
Tongren University
Tongren 554300, China.

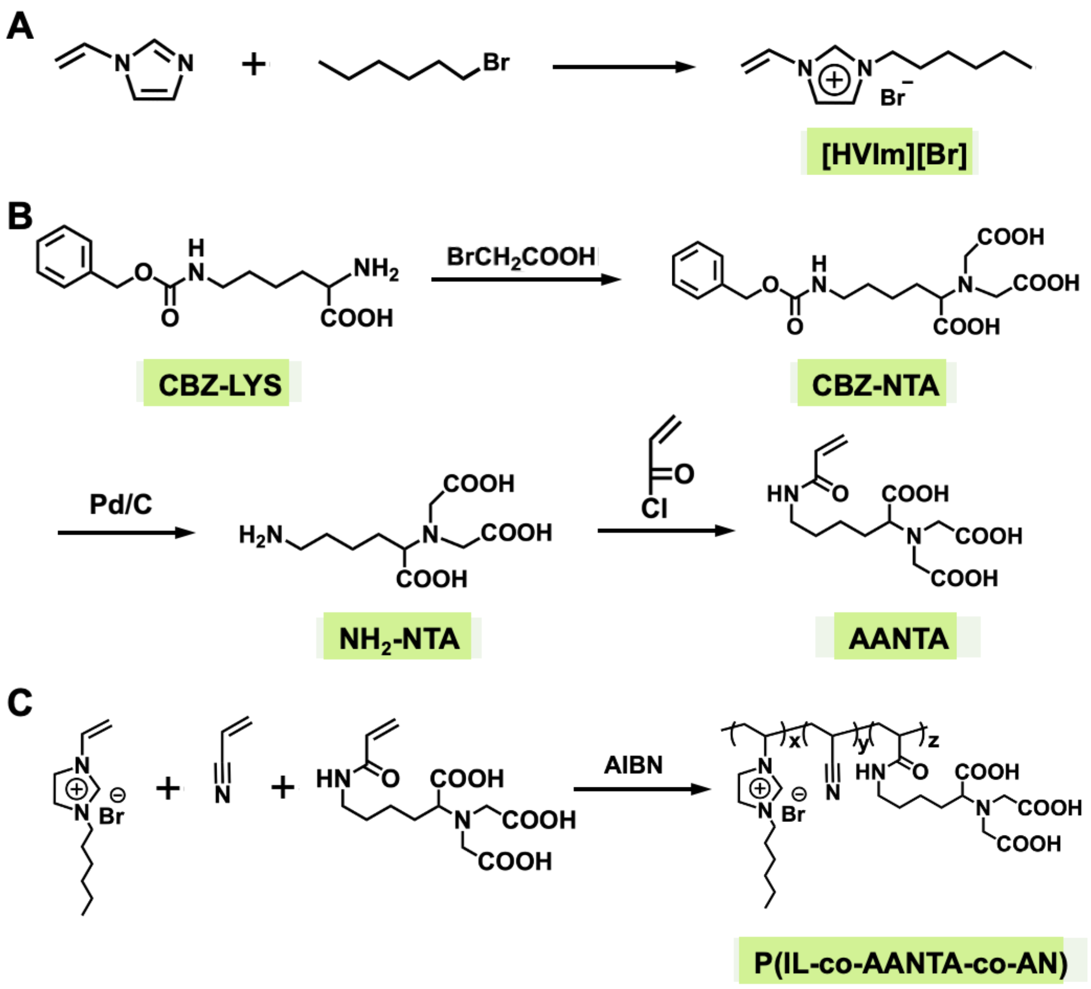

Figure S1. Synthesis scheme of A) imidazolium-type IL monomer [HVIm][Br], B) metal-ligand monomer AANTA, and C) copolymer P(IL-co-AANTA-co-AN).

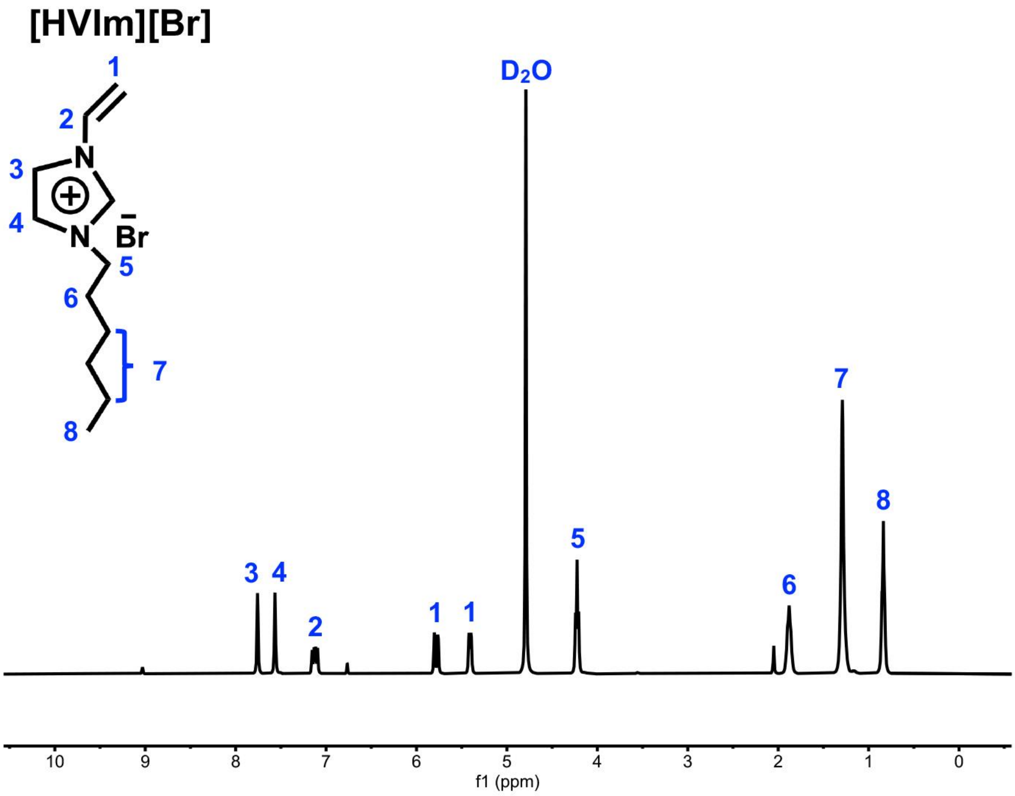

Figure S2. ^{1}H NMR spectra of [HVIm][Br].

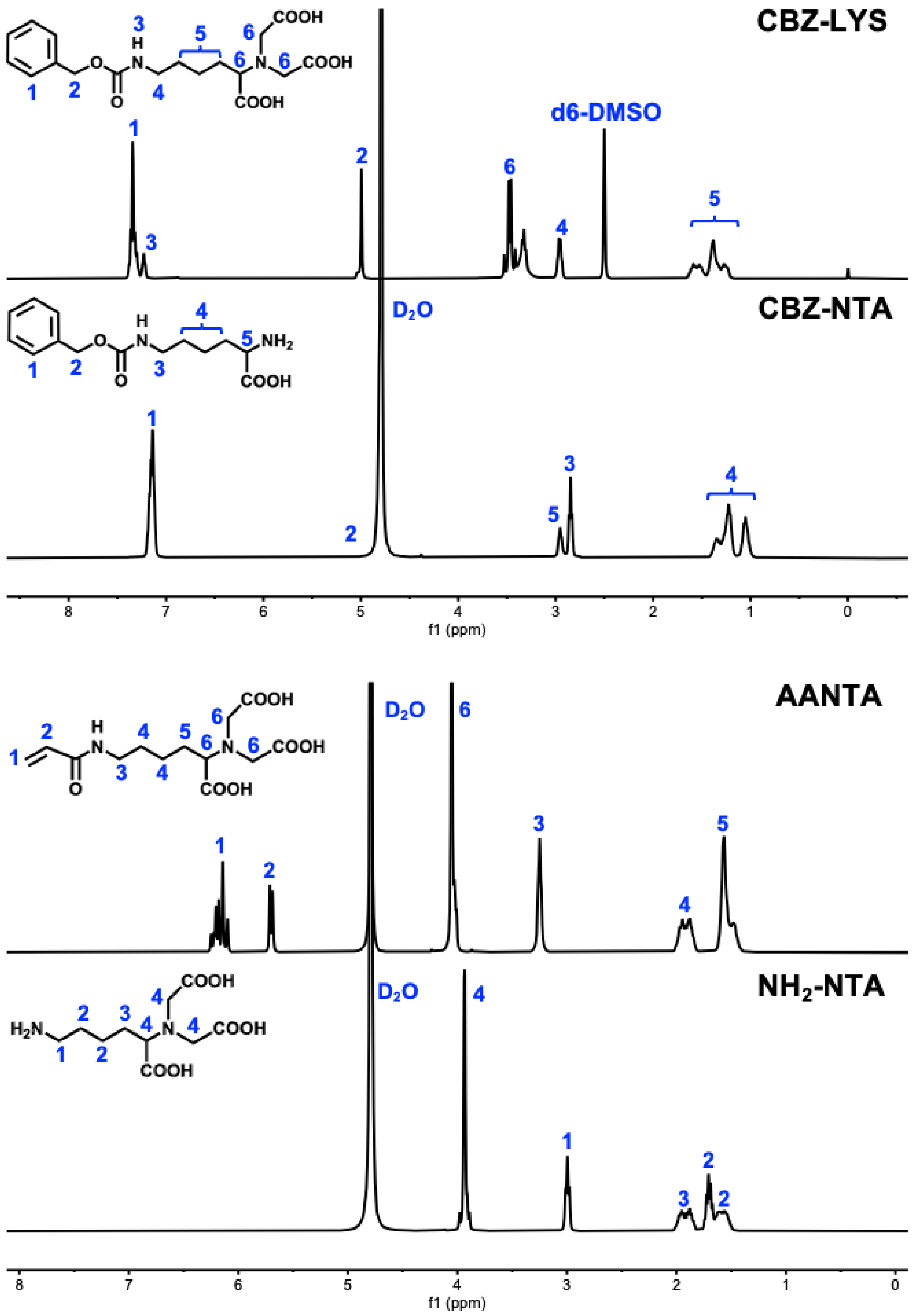

Figure S3. ^{1}H NMR spectra of CBZ-LYS, CBZ-NTA, NH_2-NTA and AANTA.

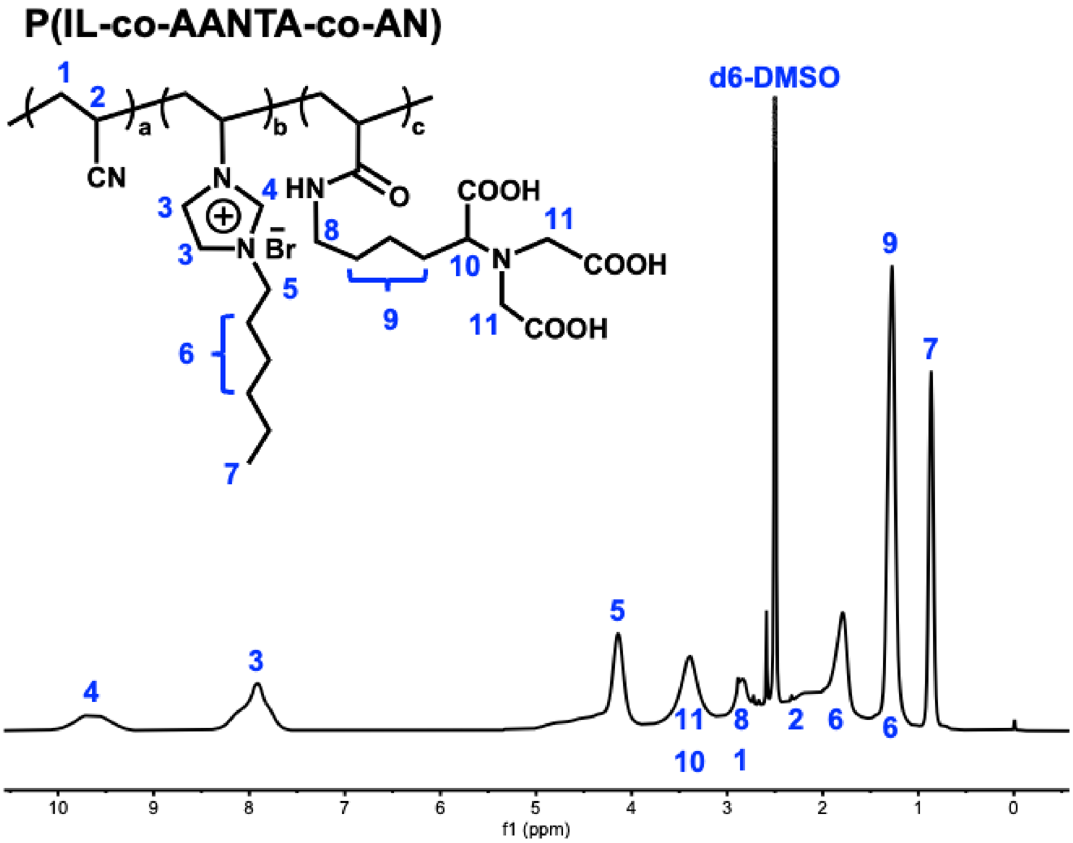

Figure S4. ^{1}H NMR spectra of P(IL-co-AANTA-co-AN).

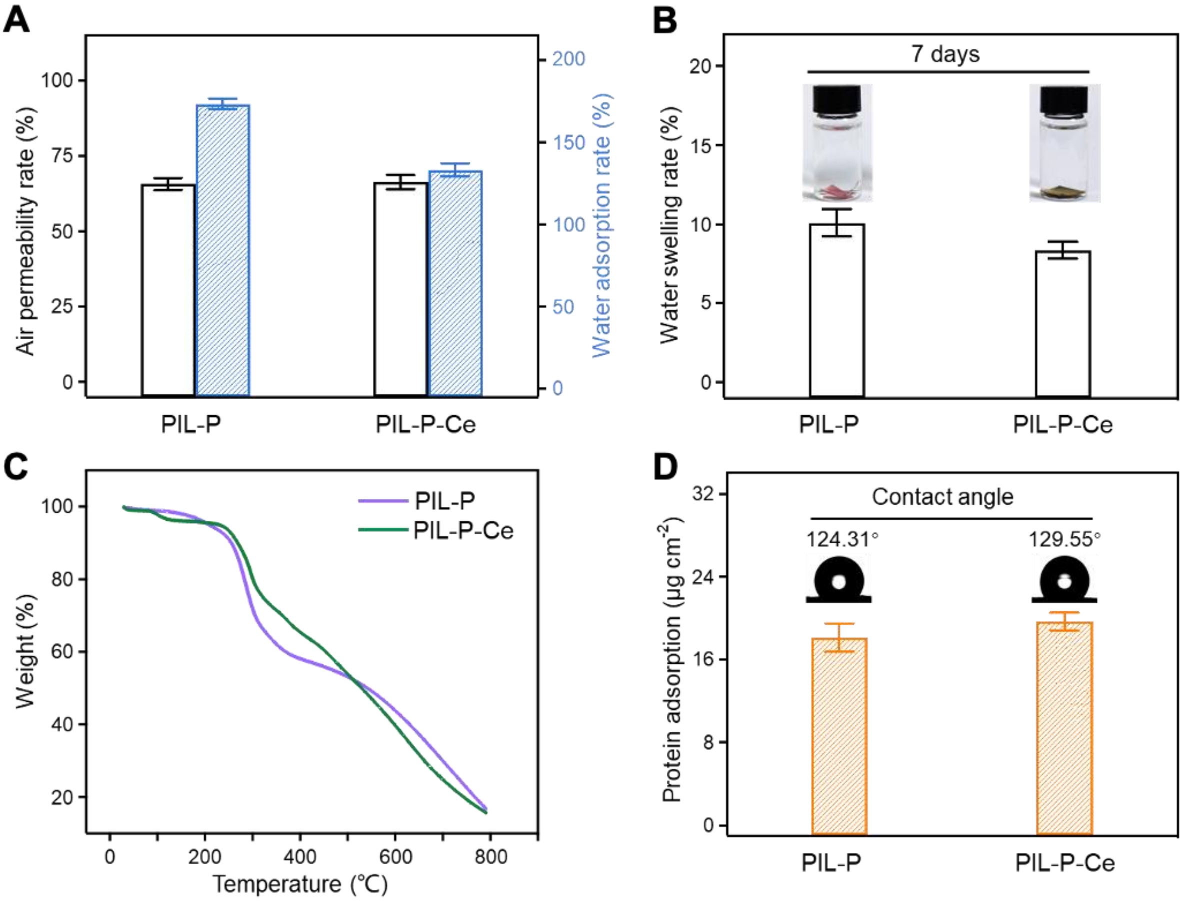

Figure S5. Physical property test of PIL-P-based membranes. A) Air permeability and water absorbability, B) water swelling rates (the inserted images show PIL-P-based membranes in PBS solution for 7 days), C) thermogravimetric analysis (TGA) curves, and D) protein absorbability and contact angles of PIL-P and PIL-P-Ce membranes. Data are mean ± s.d. N = 3.

A

	Hemolysis rate (%)	Relative growth rate (%)	
		HDF	HepG2-NTCP
PIL-P	1.18±0.51	86.47±3.81	88.74±5.01
PIL-P-Ce	1.24±0.39	88.31±4.67	92.66±3.71
PIL-P+Light	4.17±1.52	77.79±3.57	79.26±4.14
PIL-P-Ce+Light	4.32±1.26	81.16±4.63	83.08±3.81

B

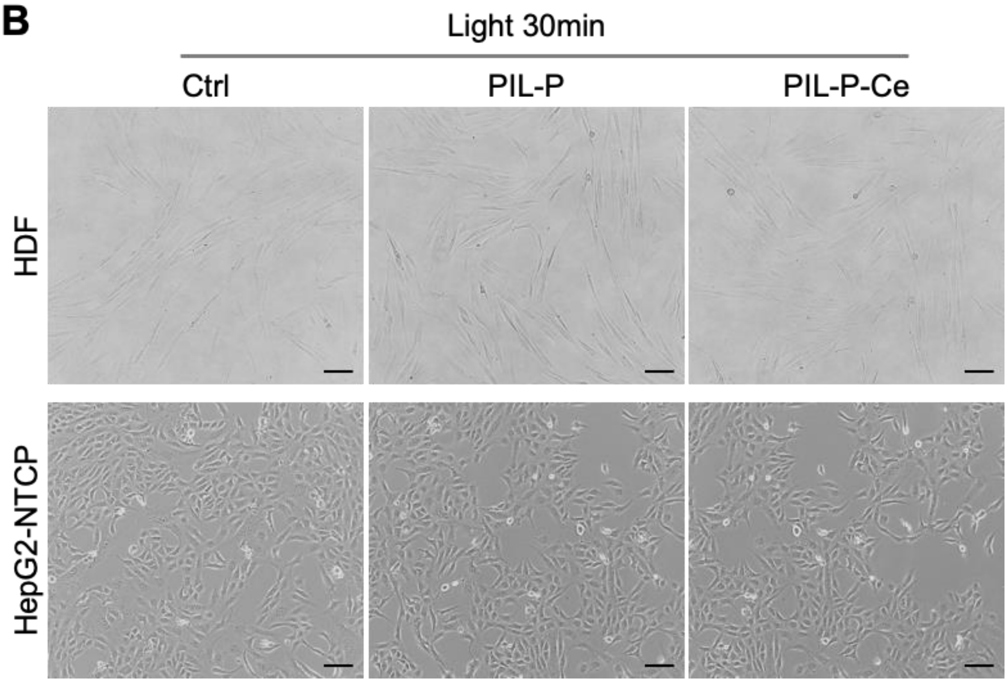

Figure S6. In vitro biocompatibility of PIL-P-based membranes. A) Relative growth rate (RGR) and hemolysis rate of PIL-P and PIL-P-Ce in the absence or presence of 650 nm light (3.5 mW cm^{-2}) for 30min. Hemocompatibility was assessed by hemolysis assays with fresh human red blood cells, and cytotoxicity by MTT assays using human fibroblasts (HDF) and human hepatocellular carcinoma cells (HepG2-NTCP). B) Images of HDF and HepG$_2$-NTCP cells after 24 h incubation with the tested membranes post 30min light treatment. PET served as control. Scale bar, 100 μm. N = 3.

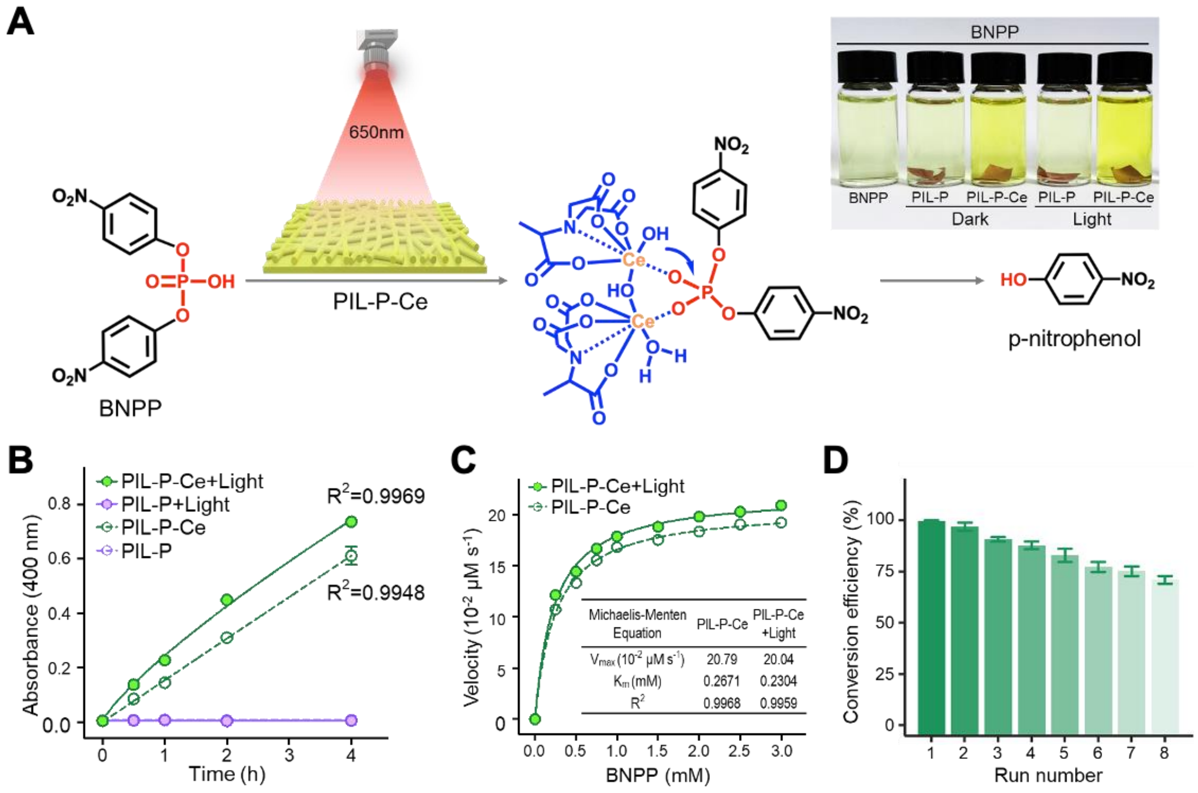

Figure S7. BNPP Decomposition by PIL-P-based membranes. A) Scheme of BNPP degradation by PIL-P-Ce under 650 nm light (3.5 mW cm^{-2}). The insert photo displays 5 mM BNPP solutions after 4 h co-incubation with tested membranes, with or without 30 min light. The blank is BNPP solution without any membrane. B) Decomposition time course of 5 mM BNPP solutions by PIL-P-based membranes with or without 30 min lighting, quantified by absorbance of the p-nitrophenol product at 400 nm. A_{400} value is proportional to p-nitrophenol concentration. C) Initial velocity for PIL-P-Ce cleavage of different BNPP concentrations, with or without 30 min light. Insert table shows reaction kinetics parameters of the Michaelis–Menten equation for each sample. D) Recycling performance test for PIL-P-Ce decomposition of BNPP under 30 min light. Data are mean ± s.d. N = 3.

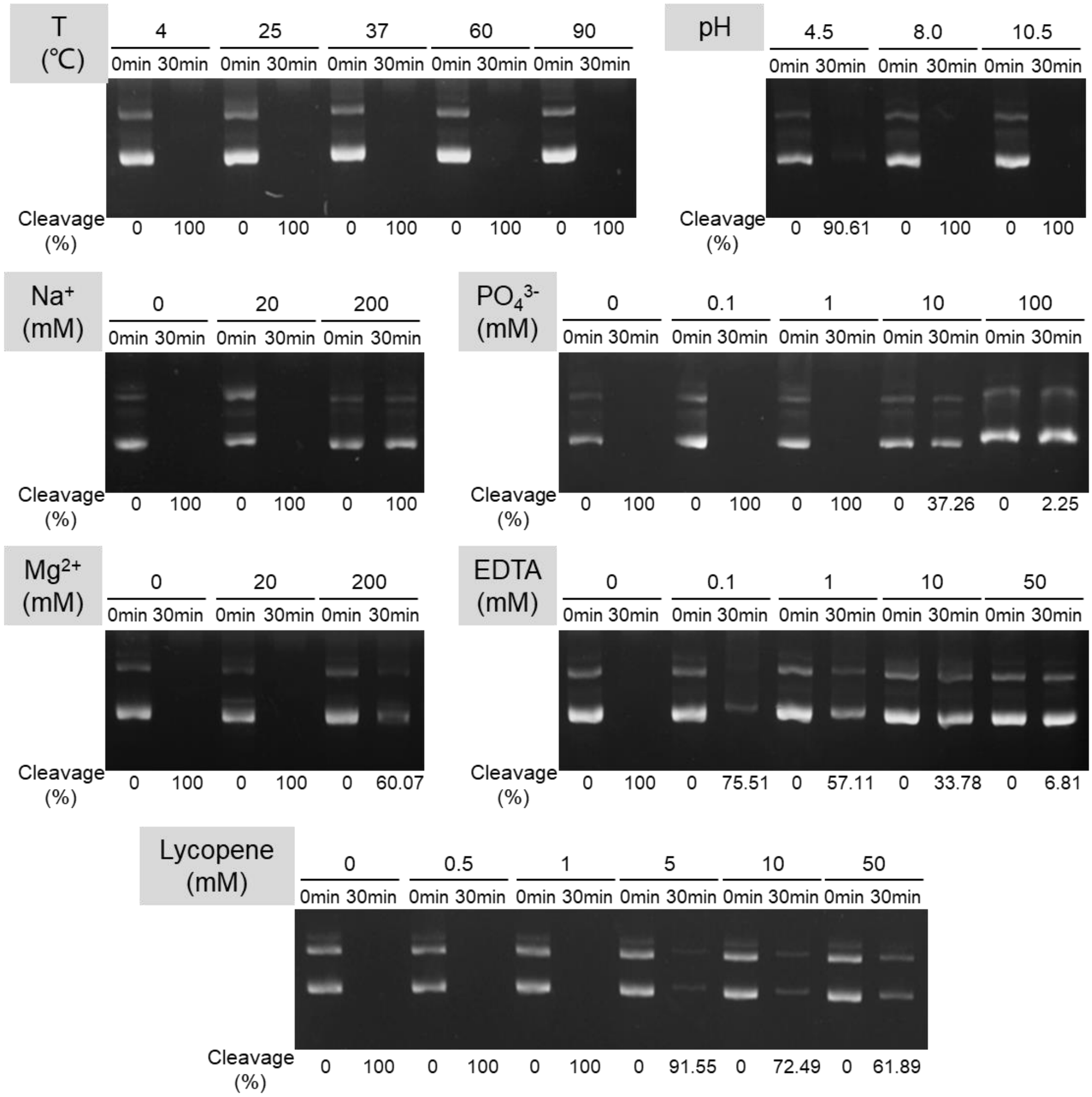

Figure S8. Impact of various factors for DNA degradation activity of PIL-P-Ce membranes. Electrophoretograms show bacterial plasmid cleavage efficiencies of PIL-P-Ce under different conditions in Figure 2G, with light for 30 min.

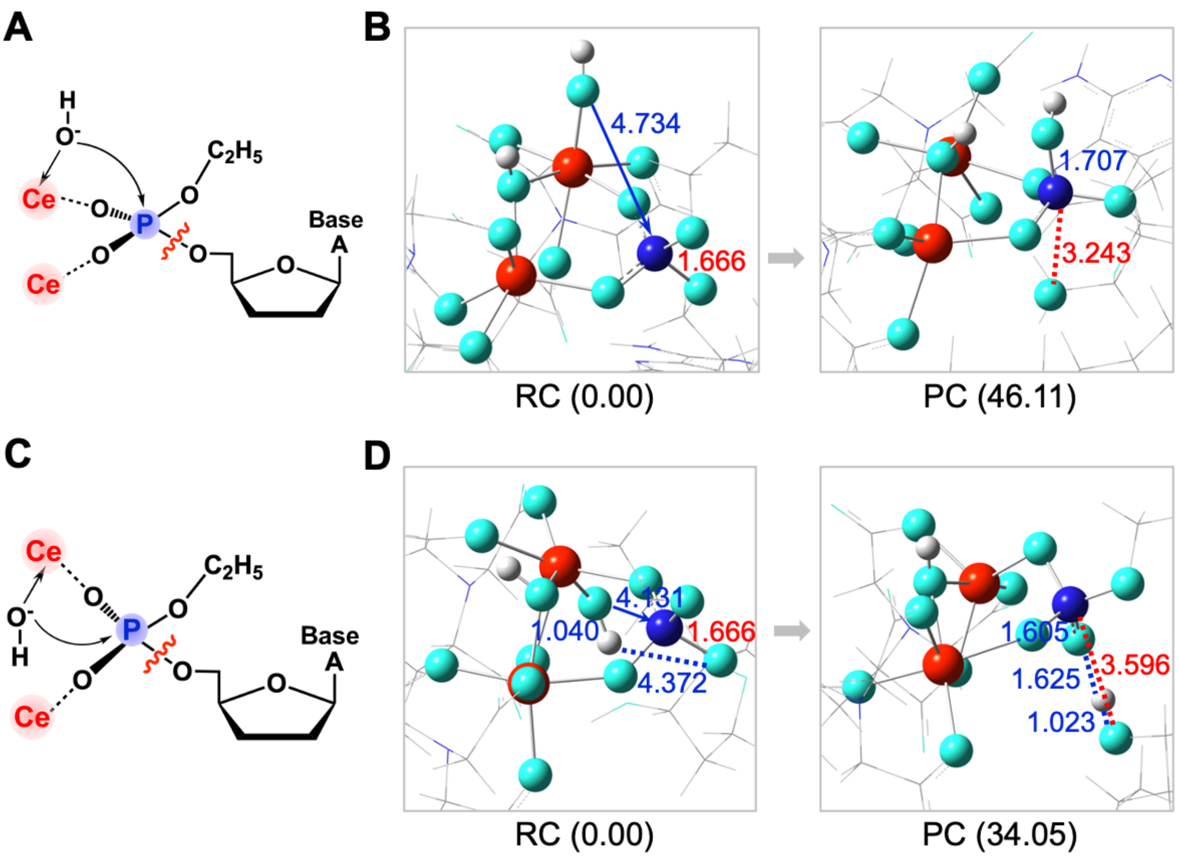

Figure S9. Intramolecular nucleophile activation mechanisms of DNA hydrolysis by Ce(IV) complex. Two computing modes for DNA hydrolysis, along with corresponding structures of reaction complex (RC) and product complex (PC) by DFT calculations are displayed in (A,B) and (C,D). Elements Ce, P, O, and H are colored with red, blue, azure, and white, respectively. Single-arrow lines indicate the reaction direction of participating groups, with red wavy lines highlighting the target P-O bond to be broken. Red dotted lines denote the length of P–O bond, blue dash lines signify distances of adjacent atoms, and other pertinent atomic distances are also marked (in Å). Bracketed values represent relative energies (in kcal/mol).

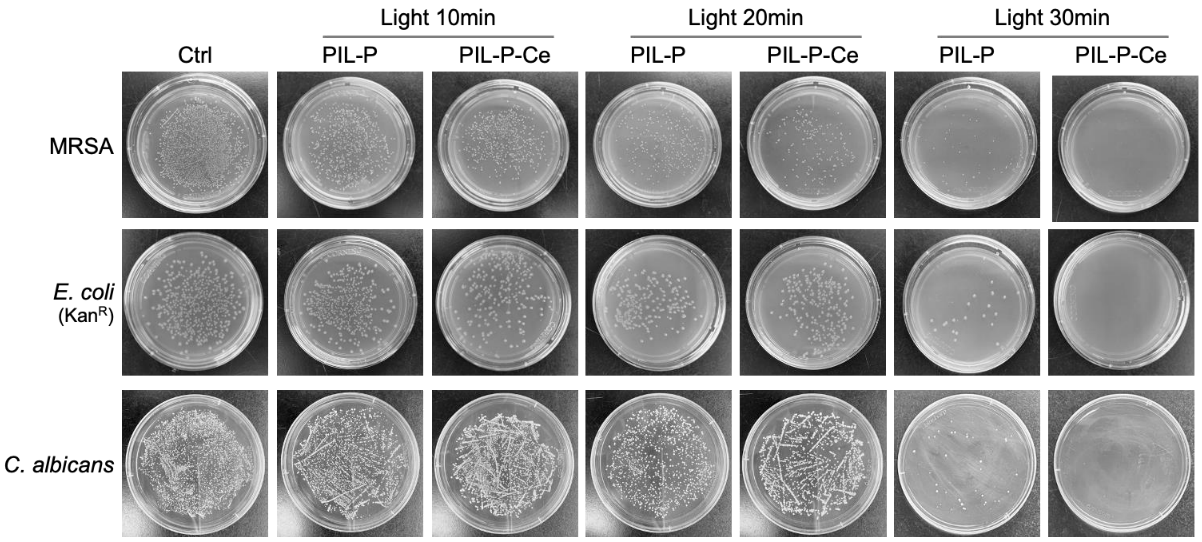

Figure S10. Antimicrobial activities of PIL-P-based membranes under light by colony assay. Three strains of MRSA, *E. coli* (Kan^{R}) and *C. albicans* were incubated with tested membranes under various exposure times to 650 nm light. PET was used as a control.

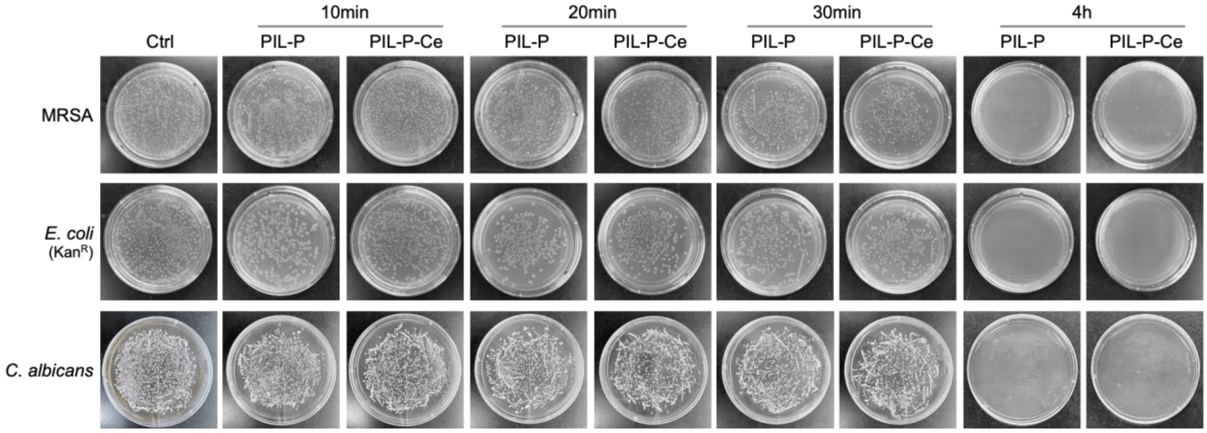

Figure S11. Antimicrobial activities of PIL-P-based membranes without light by colony assay. Three strains of MRSA, *E. coli* (Kan^{R}) and *C. albicans* were incubated with tested membranes at various exposure times without 650 nm light. PET was used as a control.

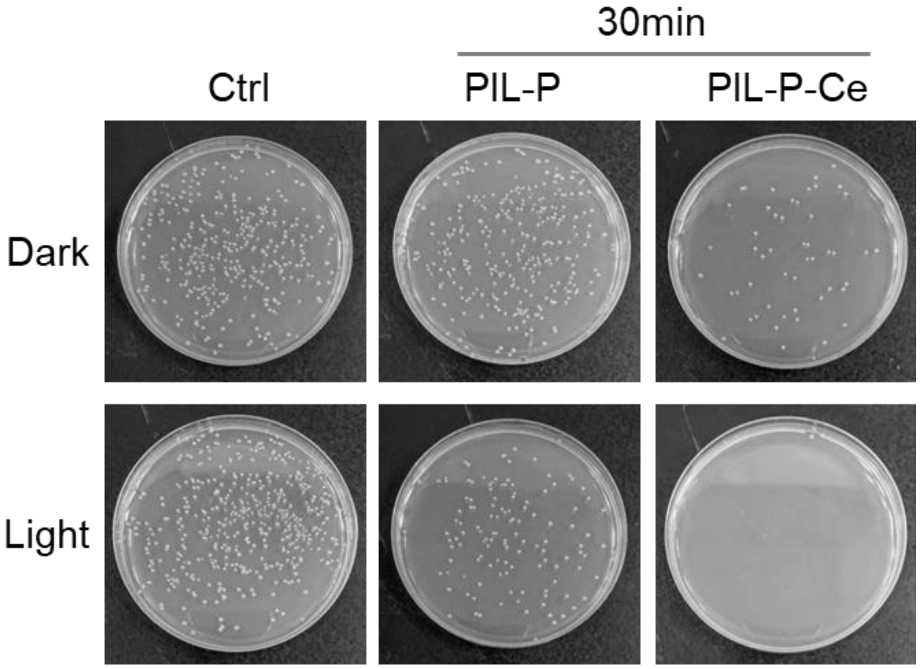

Figure S12. Transfer efficacies of ARGs in *E. coli* post-treatment with PIL-P-based membranes by colony assay. Plasmid from *E. coli* BL21 (Kan^R) was treated using PIL-P-based membranes, either with or without 30-min light exposure, and were subsequently mixed with *E. coli* DH5α (Amp^R) for transformation. Bacteria screened by dual antibiotics of kanamycin and ampicillin were counted to evaluate ARG transfer efficiencies, as presented in Figure 2H. PET was used as a control.

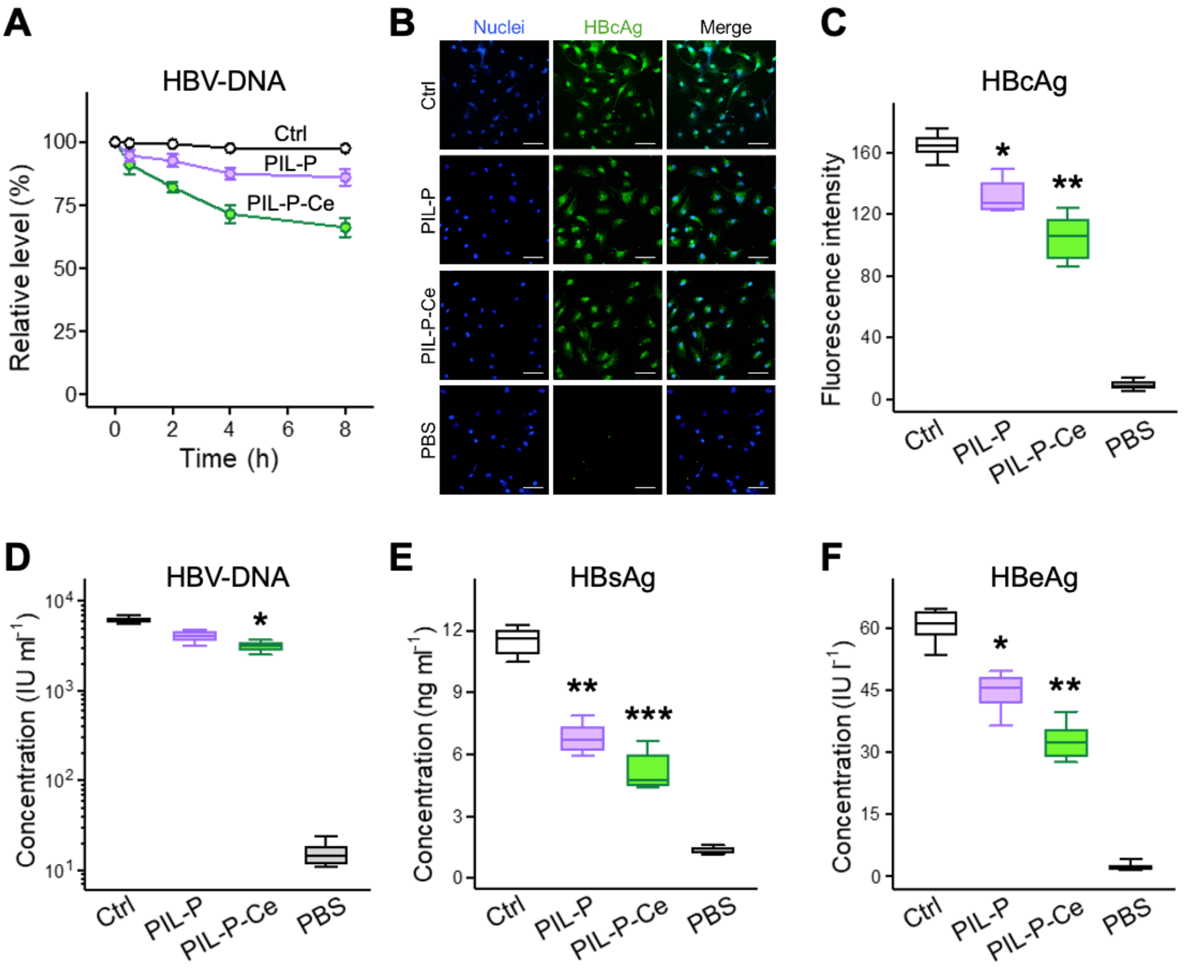

Figure S13. Anti-HBV activity of PIL-P-based membranes without light. A) Time course of HBV-DNA changes when viral sera contact control PET and PIL-P-based membranes without 650 nm light. B,C) HBcAg expression in HBV-infected hepatocytes for each group, detected *via* immunofluorescence assay without light. Scale bar, 50 µm. D–F) Levels of HBV-DNA and HBV antigens (HBsAg and HBeAg) in hepatocyte culture mediums for each group without light. PBS was used as a negative control. Data are mean ± s.d. N = 3. $*P<0.05$, $**P<0.01$, $***P<0.001$, PIL-P-based membranes are compared to PET control and analyzed by Student's *t*-test.

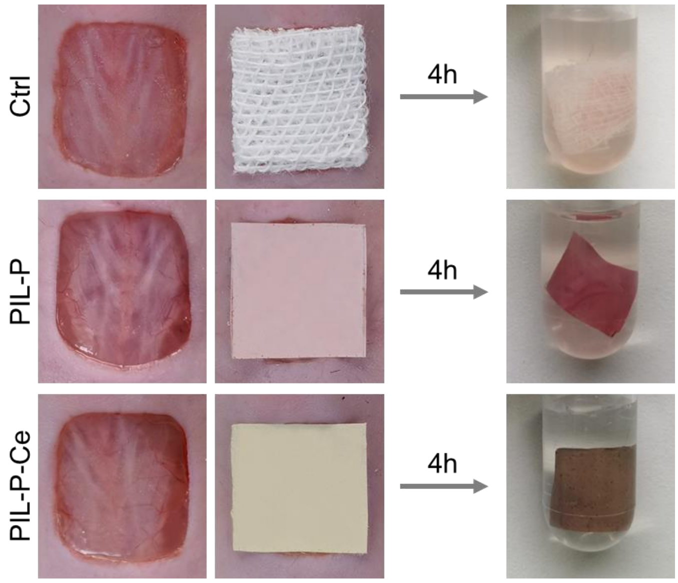

Figure S14. Images of wound treated with PIL-P-based membranes in a mouse model with bacterial-viral co-infection. Skin wounds were inoculated with a mixture of MRSA and HBV, and covered with tested PIL-P-based membranes for 4 h, with initial 30 min of 650 nm light exposure. Sterile gauze served as a control. At 4 h post application, tested membranes were immersed in sterile PBS for subsequent bacterial and viral assays.

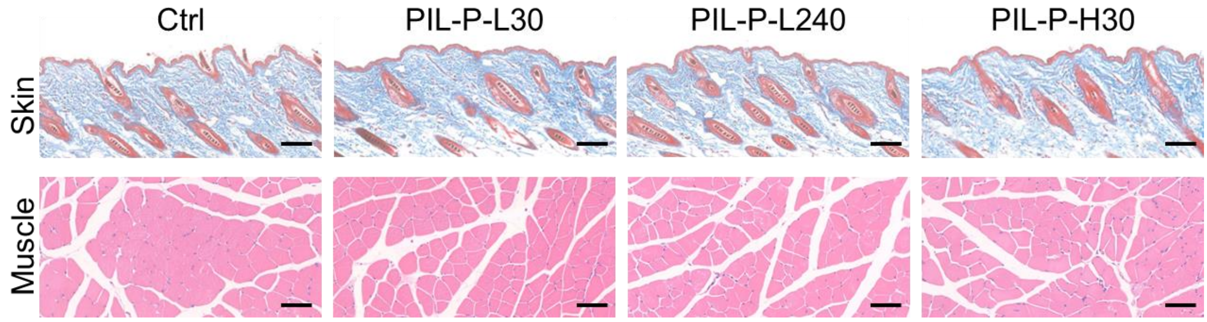

Figure S15. Representative histological images of skin (Masson staining) and muscle (H&E staining) for PIL-P groups. Mouse back wounds were covered with PIL-P membranes and subjected to various light conditions: standard treatment (3.5 mW cm^{-2} for 30 min, PIL-P-L30), extended time (3.5 mW cm^{-2} for 240 min, PIL-P-L240), and enhanced power (70 mW cm^{-2} for 30 min, PIL-P-H30). Skin and muscle tissues from wound sites underwent for histological study. Sterile gauze treated at 3.5 mW cm^{-2} for 30 min was used as control. Scale bar, 100 μm.

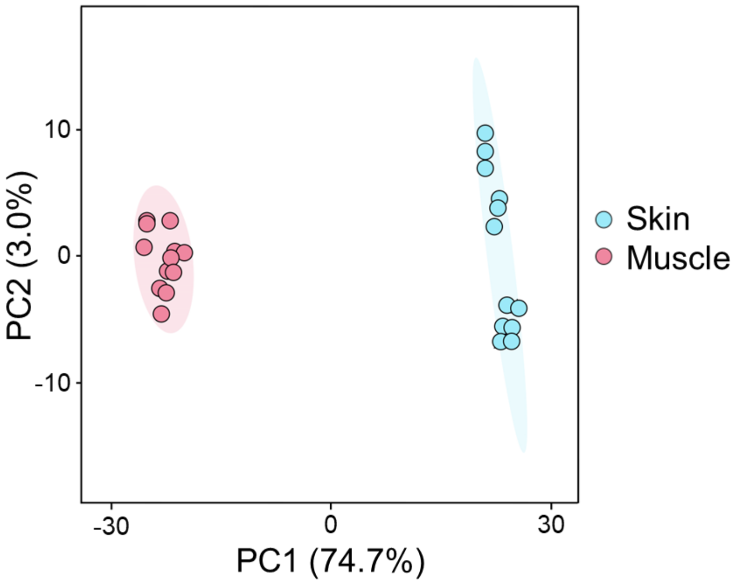

Figure S16. Principal component analysis (PCA) and heatmap of proteomic data from skin and muscle samples.

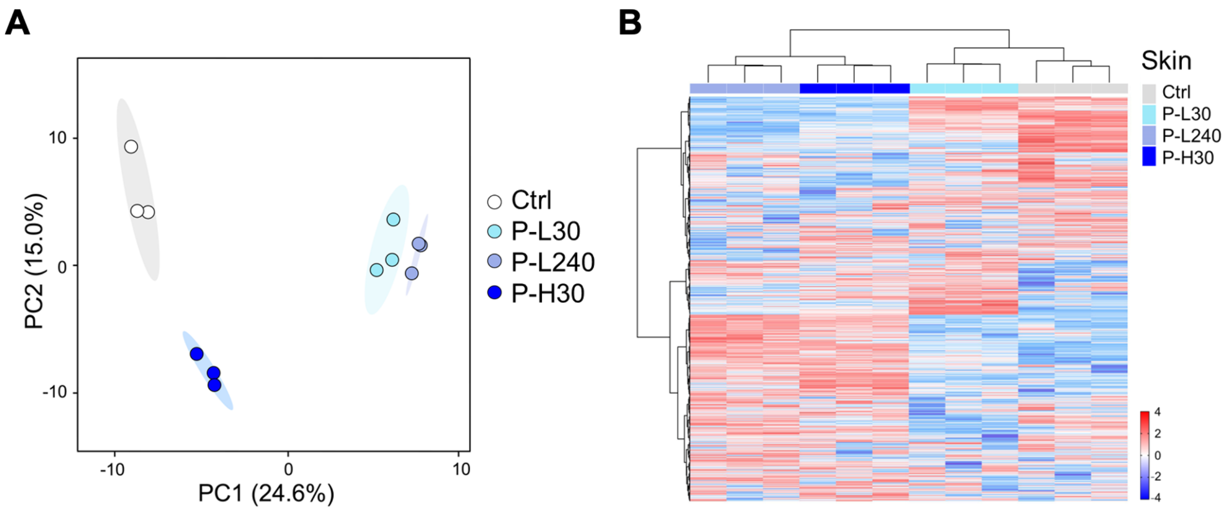

Figure S17. PCA plot (A) and heatmap (B) of proteomic data across four skin groups.

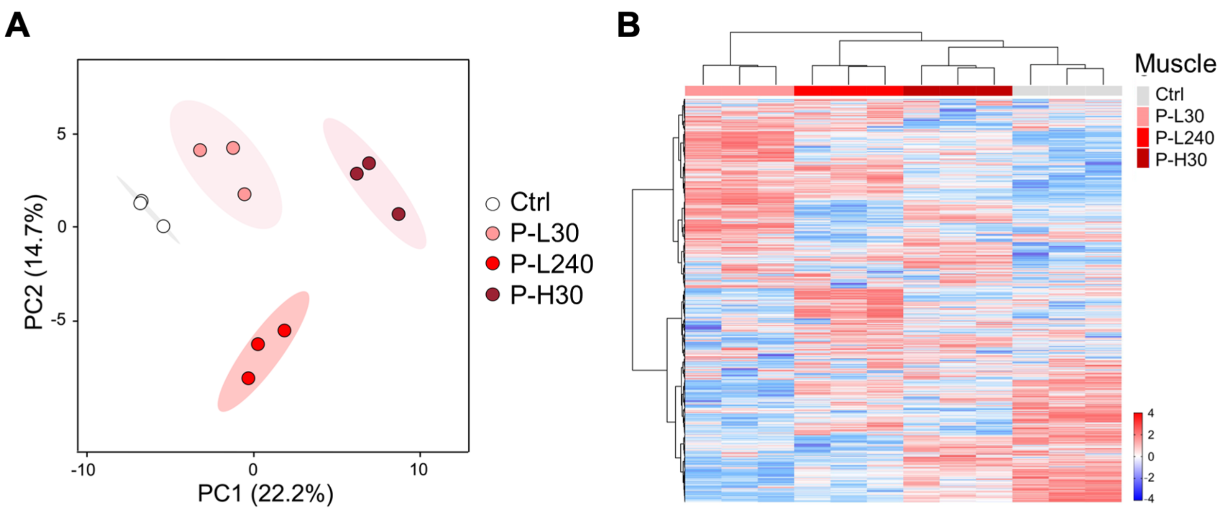

Figure S18. PCA plot (A) and heatmap (B) of proteomic data across four muscle groups.

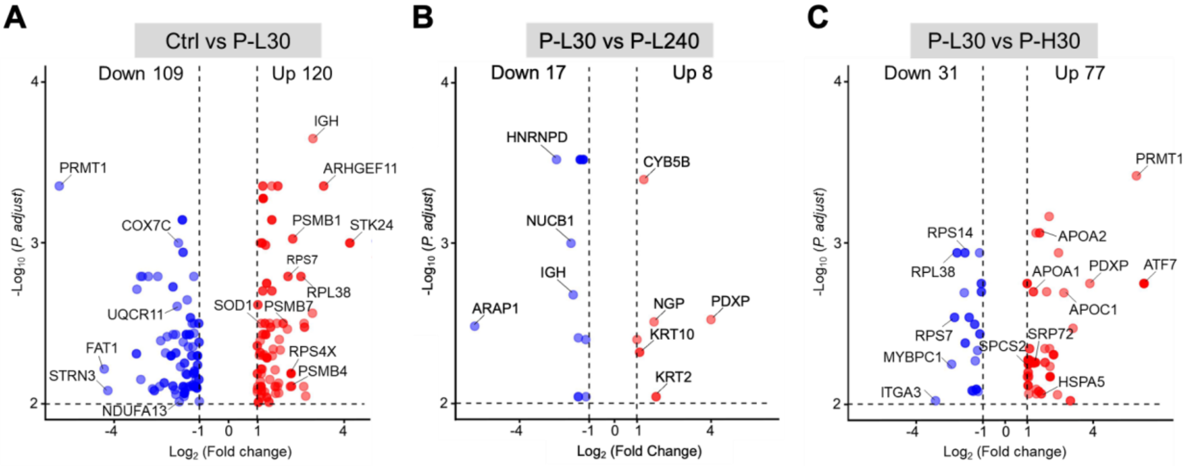

Figure S19. Volcano plots illustrating DEPs for skin comparisons between control vs. P-L30 (A), P-L30 vs. P-L240 (B), and P-L30 vs. P-H30 (C). Dotted lines indicate the selection threshold (*P. adjust* < 0.01; fold change >2 or <0.5). Red and blue dots signify upregulated and downregulated proteins, with key DEPs highlighted.

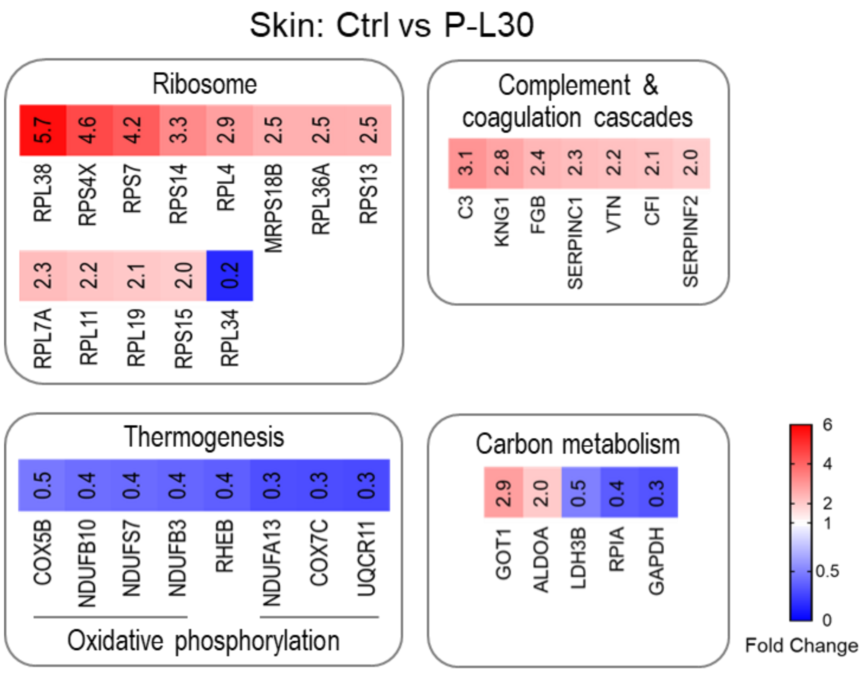

Figure S20. KEGG-enriched pathways and associated DEP fold changes for the skin comparison between control and P-L30, with the others depicted in Figure 6H.

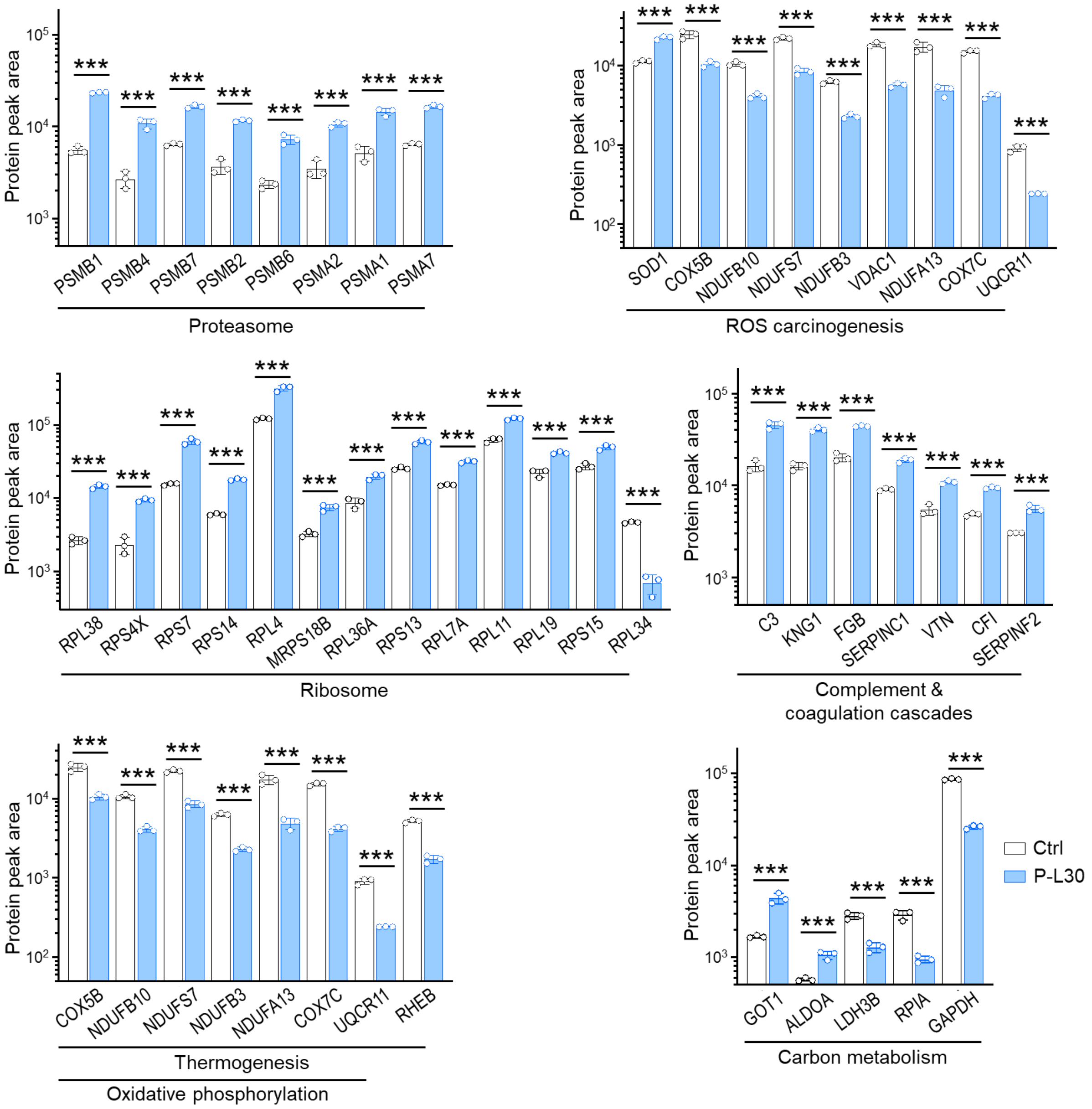

Figure S21. Peak areas of major DEPs within KEGG pathways for the skin comparison between control and P-L30. Data are mean ± s.d. N = 3. ****P*<0.001, analyzed by Student's *t*-test.

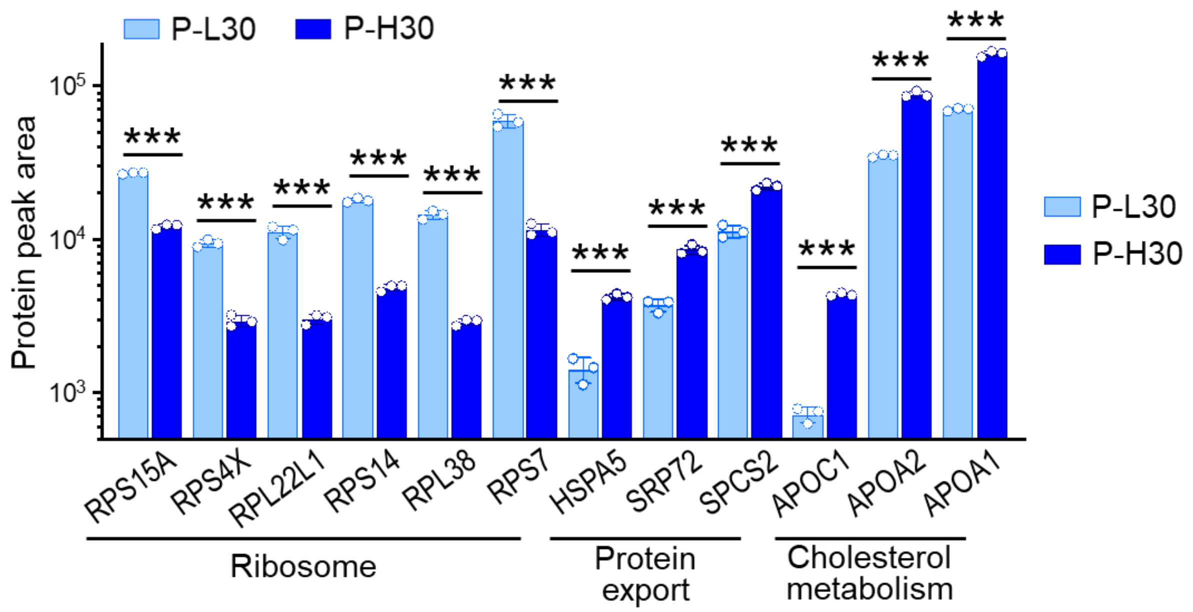

Figure S22. Peak areas of major DEPs within KEGG pathways for the skin comparison between P-H30 and P-L30. Data are mean ± s.d. N = 3. ****P*<0.001, analyzed by Student's *t*-test.

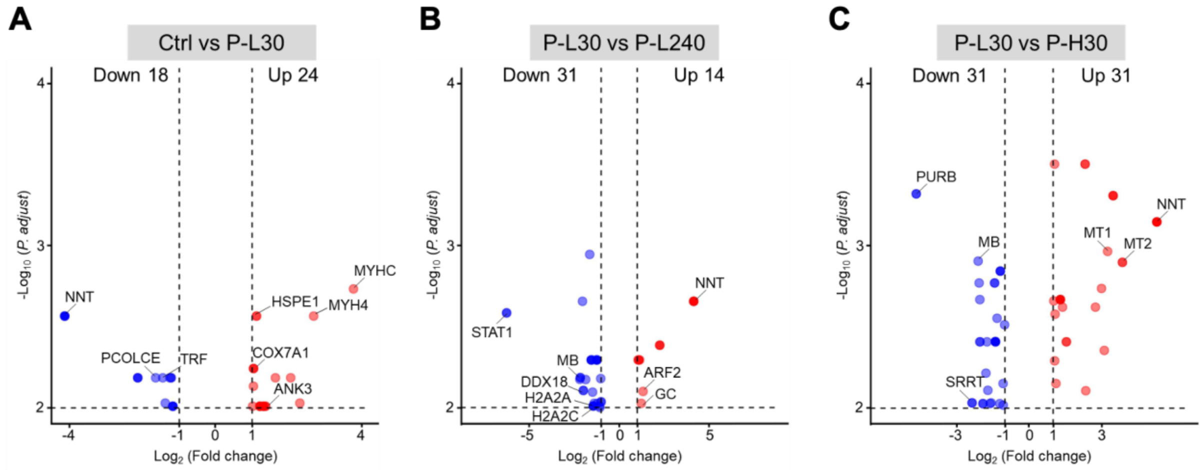

Figure S23. Volcano plots illustrating DEPs for muscle comparisons between control vs. P-L30 (A), P-L30 vs. P-L240 (B), and P-L30 vs. P-H30 (C). Dotted lines indicate the selection threshold (*P. adjust* < 0.01; fold change >2 or <0.5). Red and blue dots signify upregulated and downregulated proteins, with key DEPs highlighted.

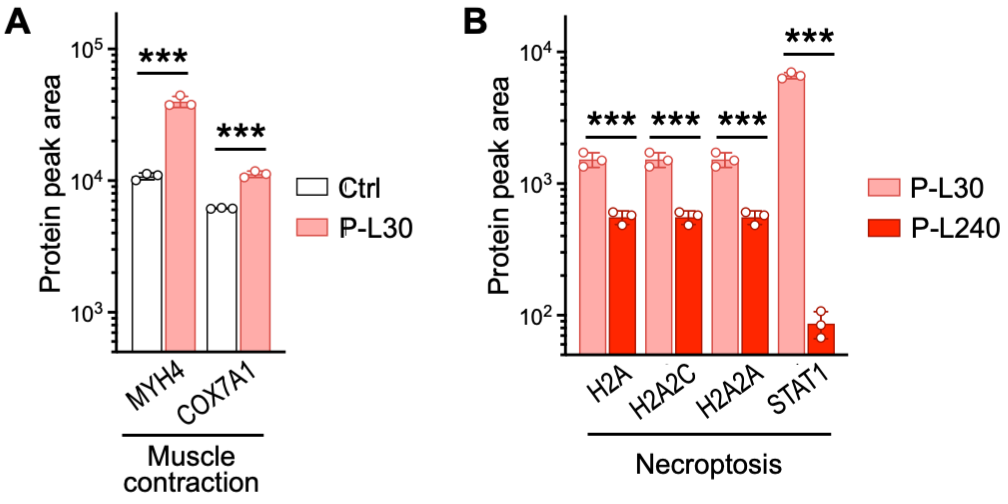

Figure S24. Peak areas of major DEPs within KEGG pathways for muscle comparisons: control vs. P-L30 (A), and P-L30 vs. P-L240 (B). Data are mean ± s.d. N = 3. ****P*<0.001, analyzed by Student's *t*-test.

Table S1. Primer sequences used for the detection of microbial ARGs in polymerase chain reaction assays.

Gene Name	Primer	Sequence	Product size (bp)
mecA	Forward	5'-GTTGTAGTTGTCGGGTTTGG-3'	298
	Reverse	5'-CTTCCACATACCATCTTCTTTAAC-3'	
Kan^R	Forward	5'-ATGAGCCATATTCAACGGGAAA-3'	816
	Reverse	5'-TTAGAAAAACTCATCGAGCATCAAATG-3'	
CDR1	Forward	5'-CCAACAATACAAGACCAGCAT-3'	566
	Reverse	5'-ACCATAGCCAATAACAACACG-3'	
Amp^R	Forward	5'-TCCCCGTCGTGTAGATCACT-3'	449
	Reverse	5'-CGGATGGCATGACGGTAAGA-3'	

Table S2. Primer sequences for qPCR assays targeting mRNA expression of selected DEPs from KEGG-enriched pathways in skin and muscle.

Gene Name	Primer	Sequence	Product size (bp)
	Forward	5'-TGTGTCCGTCGTGGATCTGA-3'	

Gapdh	Reverse	5'-CCTGCTTCACCACCTTCTTGA-3'	77
Psmb1	Forward	5'-CATCGTCGCTTCAGACACTCGA-3'	114
	Reverse	5'-CCATGGAAACCACTGCAGCCAA-3'	
Sod1	Forward	5'-GGTGAACCAGTTGTGTTGTCAGG-3'	114
	Reverse	5'-TGAGGTCCTGCACTGGTACAG-3'	
Rpl38	Forward	5'-TTCGGTCTCATCGCTGTGAGTGT-3'	195
	Reverse	5'-TCTTGACAGACTTGGCATCCTTCC-3'	
Hspa5	Forward	5'-TGTCTTCTCAGCATCAAGCAAGG-3'	144
	Reverse	5'-CCAACACTTCCTGGACAGGCTT-3'	
Apoc1	Forward	5'-CGGAACATTGGAGAGCATACCG-3'	112
	Reverse	5'-GGTCTTGGTCAAAATTTCCTTCTG-3'	
Myh4	Forward	5'-AGAGCCAAGAGGAAACTGGAGG-3'	144
	Reverse	5'-CTCGTCCTCAATCTTGCTCTGC-3'	
Stat1	Forward	5'-GCCTCTCATTGTCACCGAAGAAC-3'	123
	Reverse	5'-TGGCTGACGTTGGAGATCACCA-3'	

Movie S1. Animation for DNA hydrolysis by Ce(IV) complex as depicted in Figure 2J and 2K. The activation process of the leaving group assisted by the binding of water/hydroxide molecules to Ce^{4+} ions. The process corresponds to the transition state illustrated in Figure 2J, K. Elements Ce, P, O, and H are color-coded as red, blue, azure, and white.

Movie S2. Animation for DNA hydrolysis by Ce(IV) complex as depicted in Figure 2L and 2M. The activation process of the nucleophilic attack assisted by the binding of water molecules to Ce^{4+} ions. The process corresponds to the transition state illustrated in Figure 2L, M. Elements Ce, P, O, and H are color-coded as red, blue, azure, and white.
