# Supplementary figures and images for: Antimicrobial and Antiviral Nanofibers Halt Co‐Infection Spread via Nuclease‐Mimicry and Photocatalysis
Source: Adv Sci (Weinh). 2024 Apr 22;11(24):2309590. doi: 10.1002/advs.202309590 (PMC11200001; doi:10.1002/advs.202309590)

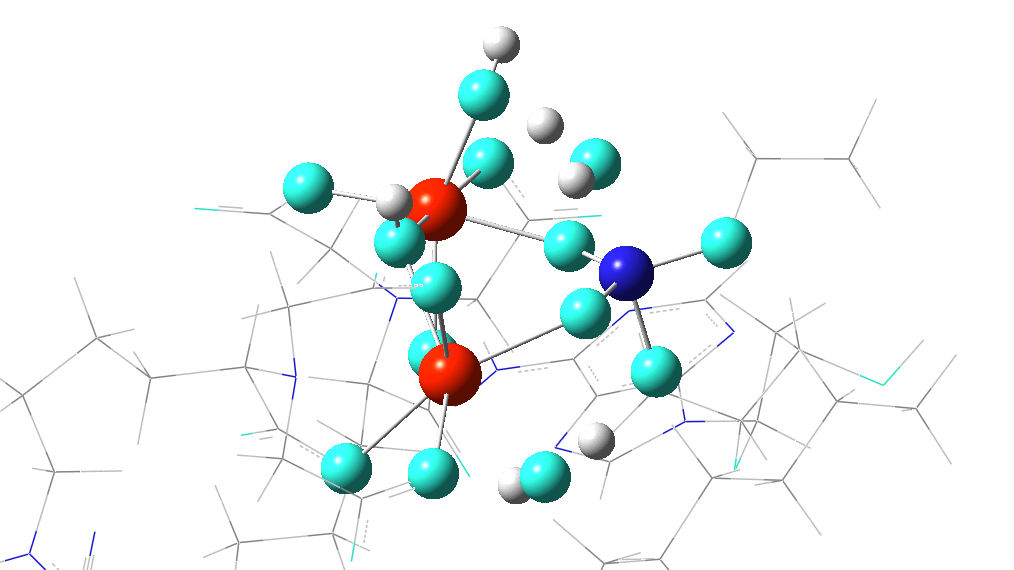

Supplement: Supplementary file 2 — Supplemental Movie 1 [file ADVS-11-2309590-s001.gif]

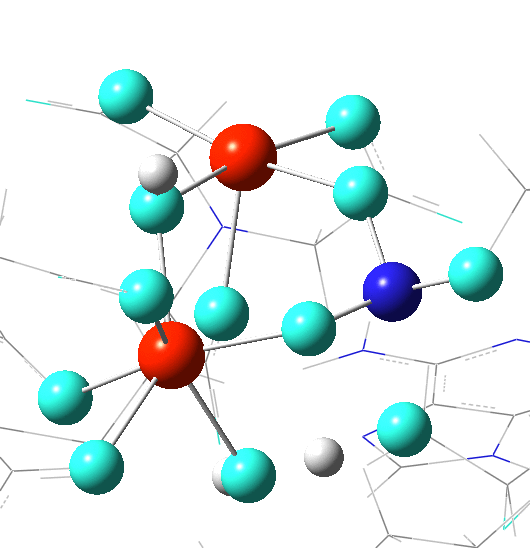

Supplement: Supplementary file 3 — Supplemental Movie 2 [file ADVS-11-2309590-s003.gif]
